# Critical Role of Perforin-dependent CD8+ T Cell Immunity for Rapid Protective Vaccination in a Murine Model for Human Smallpox

**DOI:** 10.1371/journal.ppat.1002557

**Published:** 2012-03-01

**Authors:** Melanie Kremer, Yasemin Suezer, Asisa Volz, Theresa Frenz, Monir Majzoub, Kay-Martin Hanschmann, Michael H. Lehmann, Ulrich Kalinke, Gerd Sutter

**Affiliations:** 1 Institute for Infectious Diseases and Zoonoses, University of Munich LMU, Muenchen, Germany; 2 Paul-Ehrlich-Institut, Langen, Germany; 3 Institute for Experimental Infection Research, TWINCORE, Centre for Experimental and Clinical Infection Research, a joint venture between the Helmholtz Centre for Infection Research, Braunschweig, and Hannover Medical School, Hannover, Germany; 4 Institute of Veterinary Pathology, University of Munich LMU, Muenchen, Germany; University of Alberta, Canada

## Abstract

Vaccination is highly effective in preventing various infectious diseases, whereas the constant threat of new emerging pathogens necessitates the development of innovative vaccination principles that also confer rapid protection in a case of emergency. Although increasing evidence points to T cell immunity playing a critical role in vaccination against viral diseases, vaccine efficacy is mostly associated with the induction of antibody responses. Here we analyze the immunological mechanism(s) of rapidly protective vaccinia virus immunization using mousepox as surrogate model for human smallpox. We found that fast protection against lethal systemic poxvirus disease solely depended on CD4 and CD8 T cell responses induced by vaccination with highly attenuated modified vaccinia virus Ankara (MVA) or conventional vaccinia virus. Of note, CD4 T cells were critically required to allow for MVA induced CD8 T cell expansion and perforin-mediated cytotoxicity was a key mechanism of MVA induced protection. In contrast, selected components of the innate immune system and B cell-mediated responses were fully dispensable for prevention of fatal disease by immunization given two days before challenge. In conclusion, our data clearly demonstrate that perforin-dependent CD8 T cell immunity plays a key role in MVA conferred short term protection against lethal mousepox. Rapid induction of T cell immunity might serve as a new paradigm for treatments that need to fit into a scenario of protective emergency vaccination.

## Author Summary

Prophylactic use of vaccinia virus allowed eradication of human smallpox, one of the greatest successes in medicine. However there are concerns that variola virus, the infectious agent of smallpox, may be used as bioterroristic weapon and zoonotic monkeypox or cowpox remain threatening infections in humans. Thus, new developments of safe and rapidly protecting orthopoxvirus-specific vaccines have been initiated. The candidate vaccine modified vaccinia virus Ankara (MVA) was recently shown to protect against lethal systemic poxvirus disease even when applied shortly before or after infection of mice with ectromelia virus, the probably best animal model for human smallpox. Surprisingly, little is known about the protective mechanism of early immune responses elicited against orthopoxvirus infections. Here, we used the mousepox model to analyze the immunological basis of rapidly protective MVA vaccination. In contrast to common understanding of orthopoxvirus vaccine efficacy relying mainly on antibody mediated immunity, we observed unimpaired protection also in absence of B cells. Surprisingly, rapid protection by vaccination with MVA or conventional vaccinia virus was solely dependent on T cells, irrespective of the route of injection. Thus, our study suggests a key role for T cell immunity in rapidly protective immunization against orthopoxviruses and potentially other infectious agents.

## Introduction

The most effective approach to prevent infectious diseases caused by viruses is vaccination. During the period of rational vaccine development, immunogenicity and the efficacy of vaccines were evaluated in terms of their ability to induce virus-specific antibodies. More recently however, the focus has shifted to considering the importance of cellular immune responses. In fact, vaccine-induced T cell immunity might be crucial to overcome some viral diseases. Viruses such as influenza virus or HIV are highly versatile in changing their envelope antigens to escape the host antibody response. Thus, induction of robust T cell immunity is believed to be the key to achieving successful immunization against AIDS, or enabling cross-protective capacities in next generation influenza vaccines [1]–[3]. Moreover, T cells are being recognized as playing an important role in the control of certain viral infections such as human cytomegalovirus diseases [4], [5]. Surprisingly however, there is very limited data about the contribution of T cell immunity to protection provided by any licensed viral vaccine. Even today, as in the case of influenza vaccines, most applications for marketing approval only assess the potency and efficacy of candidate vaccines using antibody correlates [6]–[8]. Moreover, regarding the immunological requirements for protective vaccination at times close to viral infection our knowledge is very limited, perhaps with exception of rabies where antibodies induced by post exposure vaccination are well known to prevent the disease and death [9], [10].

Vaccinia virus (VACV) is one of the most successful vaccines in human medicine. Vaccination of live VACV provided efficient protection against human smallpox, resulting in worldwide eradication of this devastating infectious disease [11]. Today, the development of new VACV vaccines is important due to the increasing emergence of zoonotic diseases caused by orthopoxviruses [12], and the potential misuse of these viruses as agents of bioterrorism [13]. One promising VACV vaccine candidate is based on the highly attenuated virus strain modified VACV Ankara (MVA) [14]–[16]. MVA has also been developed as a non-replicating viral vector to construct experimental recombinant vaccines against various infectious diseases [17]–[22]. Immunizations with MVA in animal models proved highly efficacious when compared to conventional VACV vaccines, and elicited antigen-specific humoral and cellular immune responses [19], [23]–[30].

Mass immunization with VACV during the smallpox eradication program indicated a critical role for cellular immune responses, since severe complications could occur in patients with T cell deficiencies [31]–[33]. However very little information is available on the role of cell-mediated immunity in protective VACV vaccination, although more recent analyses suggest that humans maintain VACV specific T cells for decades after vaccination [34]–[36].

There is more historical evidence correlating protection against smallpox with VACV neutralizing antibodies [37], [38]. Indeed, recent studies with orthopoxvirus challenge infections in animal models support the supposed protective role of antibody responses elicited by VACV immunization [39], [40]. Interestingly, very comparable levels of VACV neutralizing antibodies are found after MVA or conventional VACV (Dryvax) immunization [27]. In addition, MVA immunization can induce VACV specific antibody responses slightly earlier than conventional VACV (Elstree/Lister or Dryvax) in mice or non-human primates [39], [41]. Moreover, VACV vaccination of mice and macaques can result in full protection from lethal disease if administered shortly before or even after infection with virulent orthopoxviruses [39], [41]–[43]. In such cases rapid protection was associated with relatively high doses of vaccine, which may elicit earlier induction of antigen-specific immunity [39], [41], [43].

Here, we used ectromelia virus (ECTV) infections of mice, probably the best surrogate animal model for human smallpox. Our aim was to analyze the immunological mechanism(s) of the early protective capacity conferred by MVA immunization. Surprisingly, we found that rapid protection against lethal systemic poxvirus disease, as mediated by vaccination with MVA or conventional VACV, is solely dependent on the cellular adaptive immune response with an important role of CD4 and CD8 T cells, and perforin mediated cellular cytotoxicity. In contrast, the humoral response seems to be fully dispensable in providing early protection. Our data clearly demonstrate that T cell immunity plays a key role in the protective capacity of vaccination with a gold standard live viral vaccine. In general, the rapid induction of robust T cell responses might be of great importance for developing vaccines that need to meet the demands of protective emergency vaccination.

## Results

### Local immune responses to MVA

Intranasal (i.n.) application of MVA vaccine can rapidly induce robust protection against lethal respiratory orthopoxvirus infections [41], [43]. However, the mechanisms of protective immunity still need to be elucidated. In particular, only limited information about local immune responses in the respiratory tract is available. Recent data suggest that in the vaccinated host MVA is recognized via multiple sensor pathways, which results in the activation of innate immunity, including the synthesis of type I interferons (IFN) and chemokines. These innate responses will trigger attraction of immune cells to the site of immunization, which might be responsible for the rapid development of protective adaptive immunity [44]–[47].

**Figure 1:**
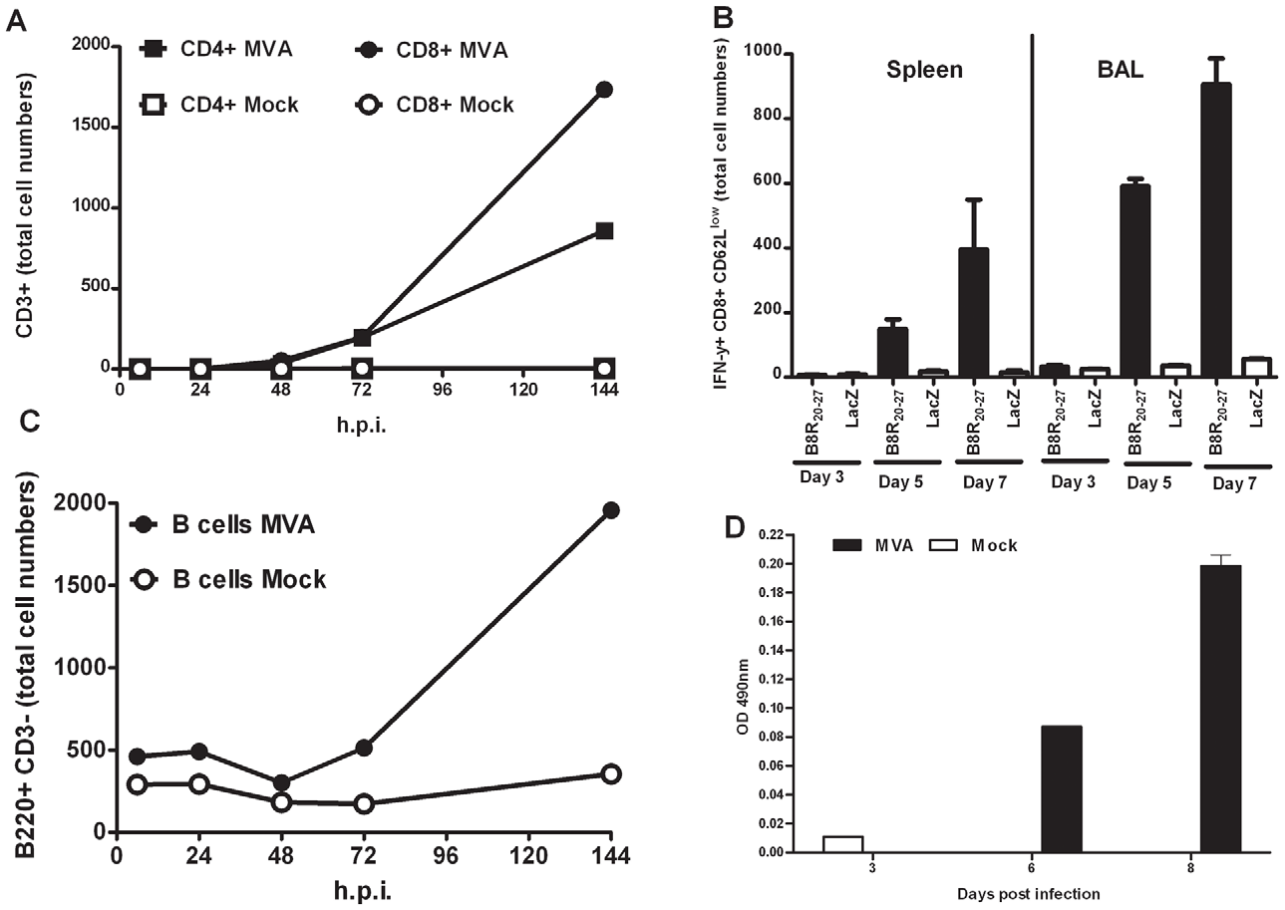
Intranasal vaccination of mice rapidly induces cellular and humoral adaptive immune responses in the respiratory tract. (A) Numbers of total CD3+ CD8+ (CD8+) and CD3+ CD4+ (CD4+) T cells in bronchoalveolar lavage (BAL) (mean pooled from three mice) at the indicated time points after inoculation with MVA (filled symbols) or mock vaccine (empty symbols). (B) Intracellular cytokine staining for gamma interferon (IFN-γ) in CD8+ CD62L^low^ spleen cells from individual mice (n = 6), or BAL cells pooled from 3 to 5 mice (mean ± SD from three independent experiments) stimulated with VACV specific B8R_20–27_ or control peptides (LacZ_876_) at 3, 5 and 7 days post MVA immunization. (C) Numbers of total B220+ CD3− B cells in BAL (mean pooled from three mice) at indicated time points after inoculation with MVA (filled symbol) or mock (empty symbol) vaccine. (D) MVA specific IgG levels in BAL fluids pooled from three mice at indicated time points after inoculation with MVA (filled bars) or mock (empty bars) vaccine. The graph depicts the OD 490 nm measured by ELISA at 1∶10 dilution of BAL fluid. All BAL data represent the results from two independent experiments.

In agreement with these data, our histopathological analysis of C57BL/6 mice two days after i.n. inoculation with MVA revealed a marked peribronchiolar and perivascular infiltrate of leukocytes in the infected lung sections compared to lung sections of mock inoculations with PBS (Figure S1). Histopathological inspection at higher magnification clearly showed neutrophil granulocytes and macrophages among the infiltrate (Figure S2). We further characterized the infiltrated leukocytes by bronchoalveolar lavages (BAL) at different time-points after i.n. administration of MVA. During the first 72 hours post infection (h p. i.) the infiltrates mainly consisted of monocytes, dendritic cells (DC), neutrophils, and NK cells (Figure S3), suggesting that the lung environment is highly favorable for antigen presentation and induction of adaptive responses. Indeed, we were able to observe increasing amounts of T and B cells in the BAL fluids of MVA exposed animals at later time points of infection (Figure 1). We detected CD4 and CD8 positive (+) T cells starting 48 h p. i., which increased in numbers to 20.5% (CD4+) and 41.5% (CD8+) of total BAL cells on day 6 p. i. (Figure 1A). To monitor VACV-specific CD8+ T cell responses we used the K^b^-restricted immunodominant determinant TSYKFESV from the VACV B8 protein being referred to as B8R_20–27_ [48] The immunodominance of B8R_20–27_ has been shown to be conserved for various orthopoxviruses including ECTV and VACV, and even in mice lacking IFN-γ or perforin [48], [49]. In MVA, the B8R open reading frame lacks some nucleotides compared to the B8R gene sequence of conventional VACV strain Lister/Elstree and encodes for a truncated B8 polypeptide. Importantly, MVA is expected to produce a fully conserved N-terminal part of the B8 protein containing the peptide epitope B8R_20–27_ and very similar expression levels of this specific B8R product were found for MVA and conventional VACV strain Elstree (Figure S4). When performing intracellular cytokine staining for interferon gamma (IFN-γ) we found proportionally high numbers of activated VACV (B8R_20–27_ epitope)-specific CD8+ T cells in BAL liquids by day 5 p. i., but comparatively lower numbers of VACV-specific CD8+ T cells in the spleen. This pattern was also observed by day 7 p. i., with about three times higher numbers of specific CD8+ T cells in BAL than in the spleen (Figure 1B). When determining B220+ CD3- B cells in the BAL cell population we could detect the presence of substantial numbers of B cells at day 6 p. i. (Figure 1C). Additionally, we also monitored for the presence of MVA-specific antibodies in BAL fluids at days 3, 6 and 8 after i.n. inoculation with MVA. We initially detected low levels of MVA-specific IgG by day 6, but antibody levels increased by day 8 p. i. (Figure 1D).

Altogether, these results indicate that early local immune responses induced after *in vivo* MVA inoculation are characterized by powerful innate responses, including the migration of massive amounts of innate immune cells to the site of infection. Moreover, antigen-specific cellular and humoral immune responses were also rapidly induced within the respiratory tract, suggesting that the rapid protection provided by MVA may be due to a close interplay between innate and adaptive immunity.

### Rapidly protective MVA immunization in the absence of NK cells or TLR, RIG-I or Mda5 mediated signaling

**Figure 2:**
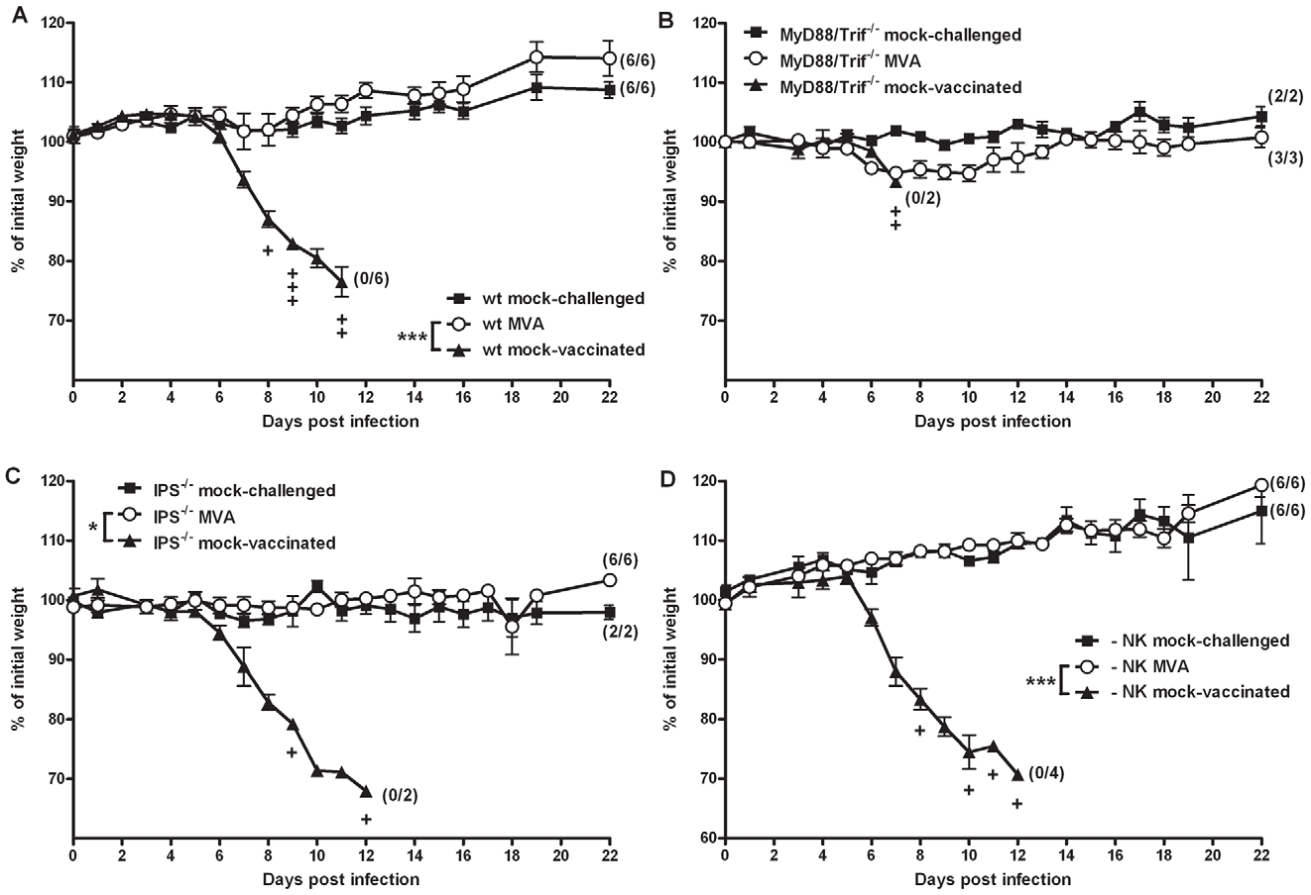
Unimpaired protective capacity of MVA immunization in mice lacking selected key adaptors of innate signaling pathways, or natural killer (NK) cells. (A) C57BL/6 mice (wt), (B) MyD88/Trif^−/−^, (C) IPS^−/−^ knock-out mice, or (D) NK cell depleted C57/BL/6 mice were challenged with 3×LD_50_ ECTV two days after MVA immunization (○), with mock-challenged (▪), and mock-vaccinated (▴) animals as controls. In all experiments weight loss of individual mice was monitored daily (n = 2 to 6 per group). +indicate the individual time of death. Error bars indicate SEMs, and the numbers of surviving/total animals are given in parentheses. Statistical significance of differences between groups is indicated by * for p-value<0.05, ** for p-value<0.01 and *** for p-value<0.001.

To elucidate the role of selected components of the innate and adaptive immune system on the rapid protection, we performed MVA immunization experiments in the C57BL/6 mouse/ECTV challenge model [41]. Briefly, we i.n. inoculated mice with 10^8^ PFU MVA two days before a lethal respiratory infection with ECTV (200 PFU corresponding to 3×LD50) (Figure 2A). This immunization fully protected the animals from disease and death after ECTV infection (P = 0.0001). In contrast, unvaccinated control mice started to show signs of morbidity (body weight loss) at about 6 days after challenge infection, and all died within 11 days (Figure 2A). Moreover, analysis of the viral loads in liver and lungs of vaccinated animals demonstrated full viral clearance at day 21 post infection (data not shown).

Recognition of invading pathogens by host cells is considered essential for activating innate and adaptive immune responses. Indeed as mentioned above, we confirmed a strong early activation of local innate immunity upon intranasal immunization (Figure S3). Also in previous experiments with IFNAR^−/−^ mice lacking the type I IFN receptor, we had found a somewhat lesser protective capacity of MVA vaccination [41], indicating a role of sensing pathways that mediate recognition signals. This sensing is mainly achieved via Toll-like receptors (TLRs) and retinoic acid-inducible gene-I (RIG-I)-like receptors (RLRs) [44].

Here, upon MVA i.n. inoculation of C57BL/6 mice we did indeed detect interleukin-6 (IL-6) and interferon-alpha (IFN-α) in BAL fluids within 6 hours and 24 hours p. i. (Figure S5) suggesting TLR and RLR dependent immune activation. We therefore performed the MVA immunization/ECTV challenge experiments in MyD88/Trif^−/−^ mice lacking TLR signaling (Figure 2B), and IPS^−/−^ mice lacking RLR signaling (Figure 2C). Clearly, the absence of either TLR or RLR signaling had no influence on the protective capacity of immunization with MVA (P = 1), confirming that several innate signaling cascades and innate immune cells can respond to MVA immunization. These likely compensate for each other in mediating sufficient immune activation to provide rapid protection.

NK cells are known to play a major role in mediating resistance to ECTV infections in mice [50]–[52] and indeed, we found NK cells infiltrating the lungs of mice early after i.n. MVA inoculation (Figure S3D). To examine the role of NK cells in rapid protection we removed NK cells by antibody-mediated depletion. Absence of NK cells on the day of MVA immunization (day -2) was confirmed by FACS analysis (Figure S6A). We observed no difference in the protective capacity acquired by NK cell-depleted mice compared to controls (Figure 2D; P = 1), indicating that the protective effect of short-term immunization with MVA is fully maintained even in the absence of NK cells.

### Rapidly protective MVA immunization in the absence of B cells

**Figure 3:**
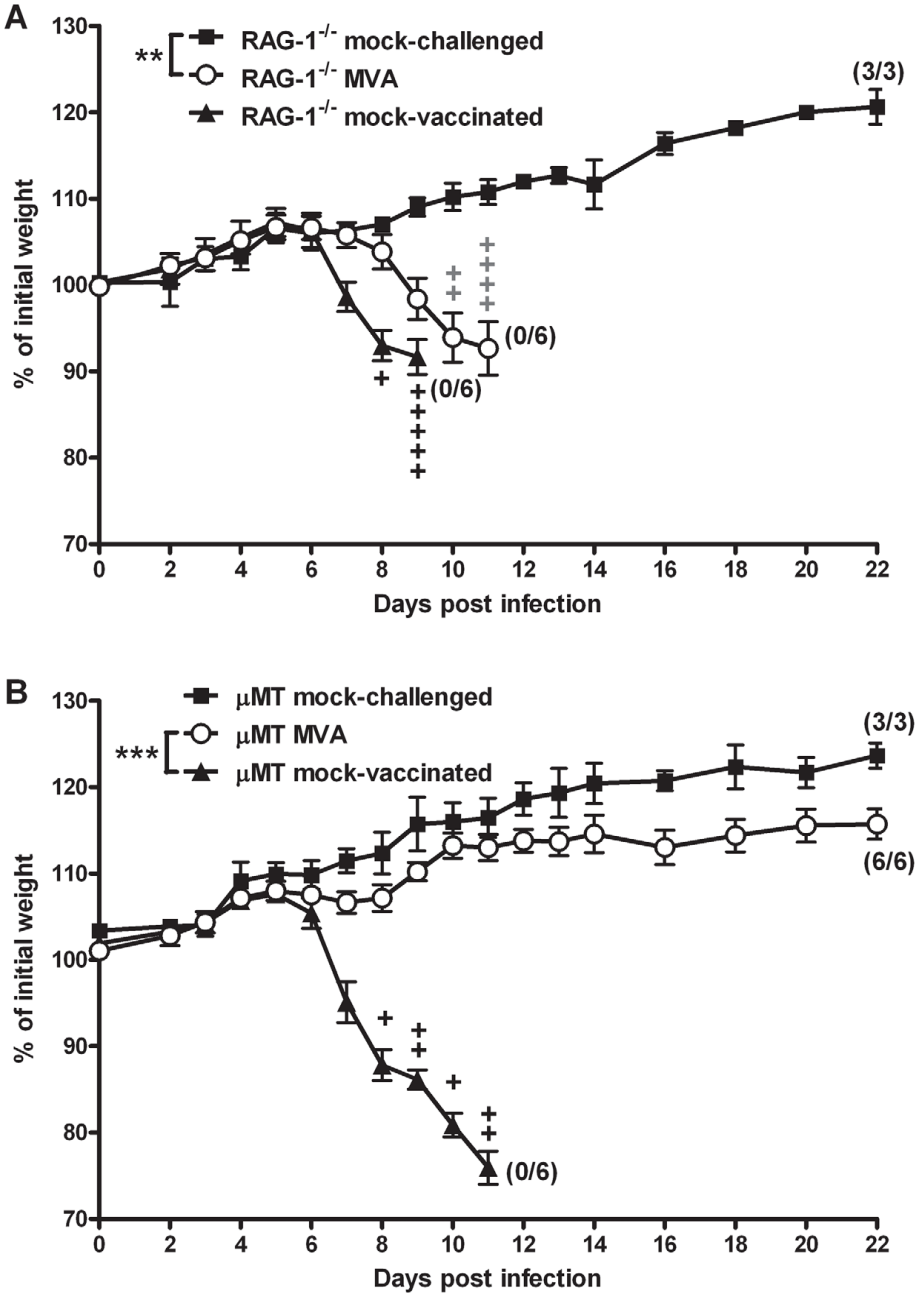
Protective immunization depends on adaptive immune responses but not on the presence of B cells. (A) RAG-1^−/−^ mice lacking mature B and T cells, or (B) B cell-deficient μMT mice were i.n. immunized with MVA (○) two days before 3×LD_50_ ECTV challenge. Mock-challenged (▪) and mock-vaccinated (▴) mice served as controls. In all experiments weight loss of individual mice was monitored daily (n = 3 to 6 per group). +indicate the individual time of death. Error bars indicate SEMs, and the numbers of surviving/total animals are given in parentheses. Data are representative of two or three similar experiments. Statistical significance of differences between groups is indicated by * for p-value<0.05, ** for p-value<0.01 and *** for p-value<0.001.

Previous work had already indicated that adaptive immunity contributes to MVA-induced protection against lethal ECTV infection [41]. Furthermore, passive immunization with vaccinia immune globulin around the day of infection fully protects mice against lethal mousepox [53], indicating that antibodies are the important players in adaptive immunity. Indeed, we showed above that specific antibodies are found in the respiratory tract early after intranasal inoculation with MVA (Figure 1D). To further analyze the role of antibody responses we performed experiments to assess the rapid protective capacity of MVA vaccination in RAG-1^−/−^ mice lacking mature T and B cells, and in B cell-deficient μMT mice [54]. Confirming previous data, vaccinated RAG-1^−/−^ mice (Figure 3A) all succumbed to the ECTV challenge infection, confirming the importance of adaptive responses in rapid protection. However, MVA immunization robustly protected B cell-deficient mice from disease and death (Figure 3B; P<0.0001). For further confirmation, we tested an alternative strain of B cell-deficient mice [55], JHT, where we also found full protective capacity of MVA immunization (Figure S7). Thus surprisingly, B cells and antibodies seem to be dispensable for this rapidly induced protective immunity.

### Rapid protection requires CD8+ and CD4+ T cells

The data so far indicated that T cells might play a crucial role in short-term protective immunity. Analysis of VACV specific CD8+ T cell responses elicited by MVA immunization in JHT mice showed levels of VACV specific CD8+ CD62^low^ T cells comparable to those induced in C57BL/6 wt mice (Figure S8). Thus, B cell-deficient mice are still able to mount specific T cell responses, further corroborating that T cell responses might be important for short-term protection.

**Figure 4:**
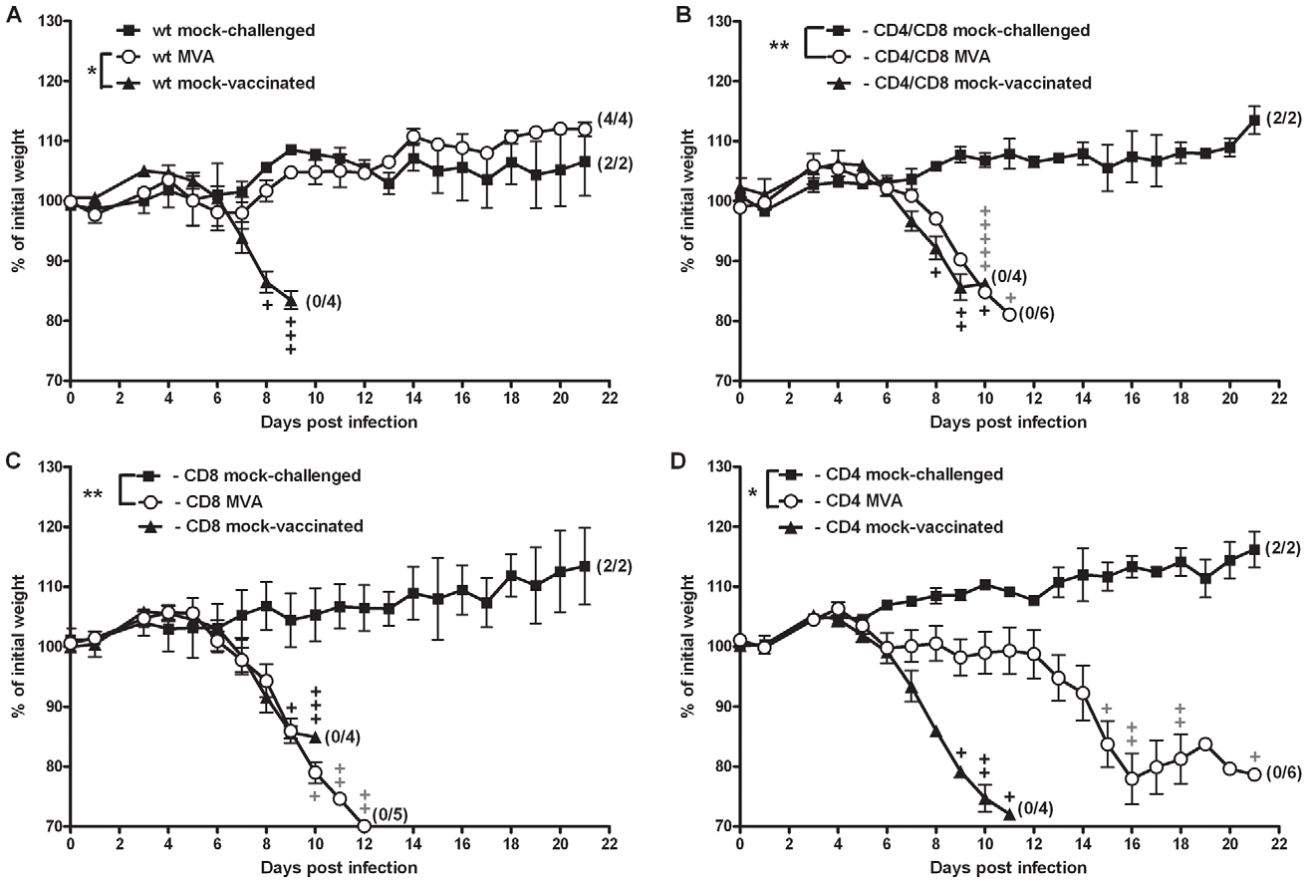
T cells are required for rapidly protective immunization. (A) C57BL/6 mice (wt), (B) mice depleted of CD4+ and CD8+ T cells, (C) depleted of CD8+ T cells, or (D) depleted of CD4+ T cells were challenged with 3×LD_50_ ECTV two days after MVA immunization (○), with mock-challenged (▪) and mock-vaccinated (▴) mice as controls. In all experiments weight loss of individual mice was monitored daily (n = 2 to 6 per group). +indicate the individual time of death. Error bars indicate SEMs, and the numbers of surviving/total animals are given in parentheses. Data are representative of two or three similar experiments. Statistical significance of differences between groups is indicated by * for p-value<0.05, ** for p-value<0.01 and *** for p-value<0.001.

**Figure 5:**
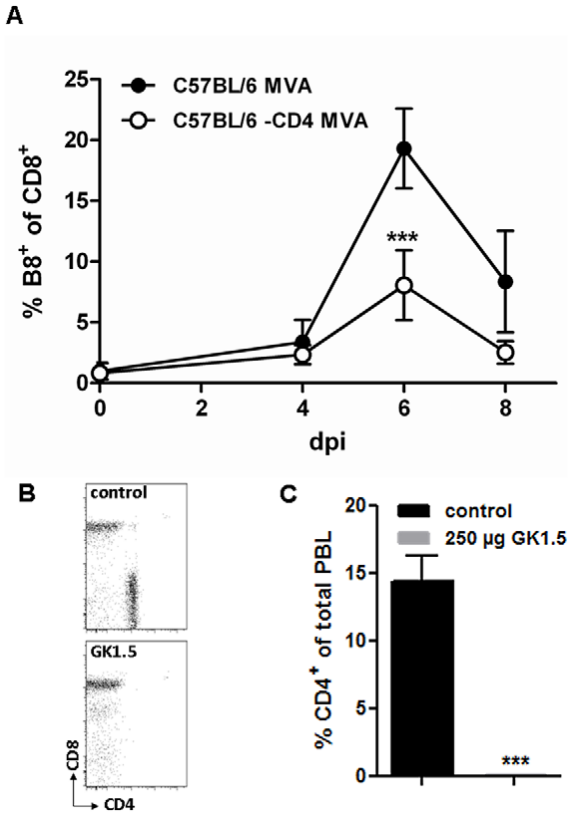
CD4+ depleted mice show a reduced CD8+ virus-specific T cell expansion upon MVA immunisation. C57BL/6 mice were treated twice with 250 µg anti-CD4 Ab (GK1.5 Harlan) two and one day prior to immunization with 2×10^5^ pfu MVA. As control untreated mice were immunized with 2×10^5^ pfu MVA. (A) The frequency of expanded B8R-specific CD8+ T cells in control mice (black) and CD4-depleted mice (white) was monitored within the blood at different time points post MVA immunization. (B) CD4+ T cell depletion efficiency was monitored at the day of immunization by flow cytometry of blood samples. CD4+ and CD8+ T cells within CD3+ population of blood lymphocytes are shown from representative mice. (C) CD4+ T cell depletion efficiency at 6 dpi, the day of maximal T cell expansion, was calculated as % CD4+ of total peripheral blood lymphocytes (PBL). Data shown are pooled from two independent experiments with n = 5 mice per group. Statistical significance is shown by *** p>0.0001 using two-tailed t-test.

Thus, we depleted C57BL/6 mice of CD4+ T cells, CD8+ T cells or both T cell subsets by i.p. injection of specific antibodies, and confirmed successful depletion by FACS analysis at the time point of MVA immunization (day 0) and at day 7 after vaccination (Figure S6B,C; data not shown). Control C57BL/6 mice were again fully protected by MVA immunization two days prior to the lethal respiratory challenge infection with ECTV (Figure 4A). In contrast, vaccinated mice depleted of CD8+ T cells, or both T cell subsets (Figure 4B,C) succumbed to ECTV infection, with similar disease pattern and time to death as compared to unvaccinated animals. On the other hand, depletion of CD4+ T cells (Figure 4D) prior to MVA immunization resulted in delayed onset of disease, since the start of striking body weight loss occurred about six days after the onset of symptoms in unvaccinated controls. Nevertheless, CD4+ T cell depletion in immunized animals also resulted in 100% mortality within 21 days post challenge. These data clearly suggested that both CD4+ and CD8+ T cells are required to rapidly protect mice by MVA vaccination. To assess the need for CD4+ T cells in some more detail, we analyzed CD4-depleted C57BL/6 mice for defects in mounting VACV-specific antibodies or CD8+ T cells following MVA immunization. Lack of CD4+ T cells resulted only in a minor reduction of IgG antibody responses as revealed by ELISA testing of sera at day 21 post vaccination with 10^8^ PFU MVA (i.n.). The CD4 cell depleted mice were clearly able to mount levels of VACV-specific antibodies (mean titer of pooled sera 1280) that were just about two fold lower than responses obtained in control mice (data not shown). To study the impact of CD4+ T cell depletion on MVA induced CD8+ T cell responses we monitored for the expansion of endogenous CD8+ T cells specifically recognizing the B8R_20–27_ epitope (TSYKFESV) by FACS analysis using a TSYKFESV-Kb pentamer (ProImmune) (Figure 5). Upon inoculation of C57BL/6 mice with 2×10^5^ PFU MVA, B8R-specific T cells massively expanded and reached a maximum of approximately 20% of total CD8+ T cells at day 6 after infection. On the contrary, MVA-vaccinated CD4+ depleted mice showed a significantly reduced CD8+ T cell expansion reaching less than 10% of total CD8+ T cells (Figure 5A). This data suggested that CD4+ T cells seem to play an important role in regulating the strength of the MVA induced CD8- T cell response.

**Figure 6:**
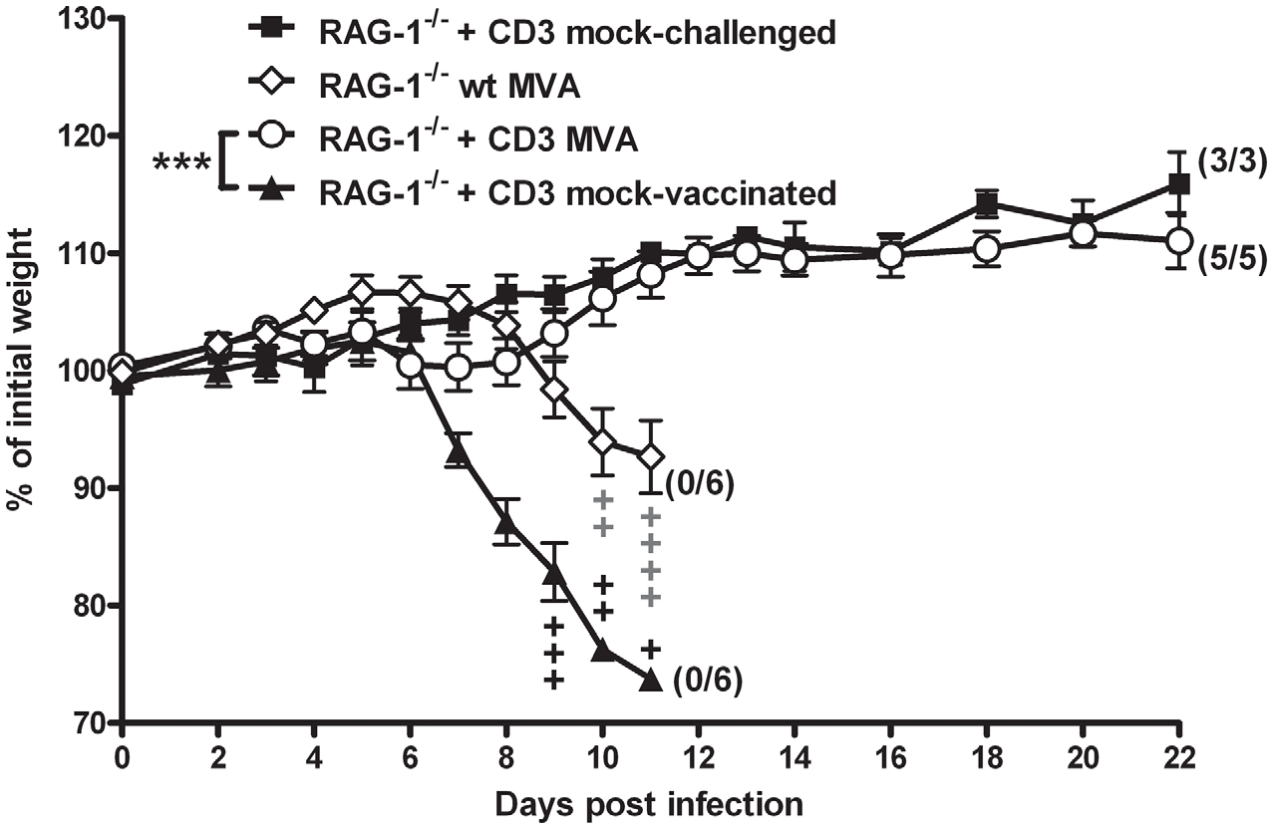
Transfer of naïve CD3+ T cells into RAG-1^−/−^ mice restores rapid protection by MVA vaccination. Vaccinated RAG-1^−/−^ mice (◊) or vaccinated RAG-1^−/−^ mice that received naïve CD3+ spleen cells two days before immunization with MVA (○) were challenged with 3×LD_50_ ECTV two days after vaccination. Groups of RAG-1^−/−^ mice that received naïve CD3+ spleen cells served as mock-challenged (▪) or mock-vaccinated (▴) controls. In all experiments weight loss of individual mice was monitored daily (n = 3 to 6 per group). +indicate the individual time of death. Error bars indicate SEMs, and the numbers of surviving/total animals are given in parentheses. Data are representative of two similar experiments. Statistical significance of differences between groups is indicated by * for p-value<0.05, ** for p-value<0.01 and *** for p-value<0.001.

To further confirm the relevance of T cell immunity in rapid protection by MVA immunization we adoptively transferred naïve CD3+ splenocytes, which comprise mainly T cells, into RAG-1^−/−^ mice. After two days these mice were vaccinated with 10^8^ PFU MVA and challenged two days later with ECTV. Indeed, the transfer of T cells prior to vaccination was fully sufficient to protect RAG-1^−/−^ mice against lethal infection, whereas control RAG-1^−/−^ mice all died despite MVA immunization (Figure 6; P = 0.0003). Interestingly, control RAG-1^−/−^ showed a slight delay in onset of disease upon MVA immunization (Figure 3A, Figure 6) which cannot be observed in mice depleted of CD4+ and CD8+ T cells (Figure 4). This effect may be due to elevated NK cell numbers in non-lymphoid tissue of RAG-1^−/−^ mice [56] that might transiently compensate for the lack of T and B cells.

### Cytotoxic effector molecule perforin is essential for rapid protection

**Figure 7:**
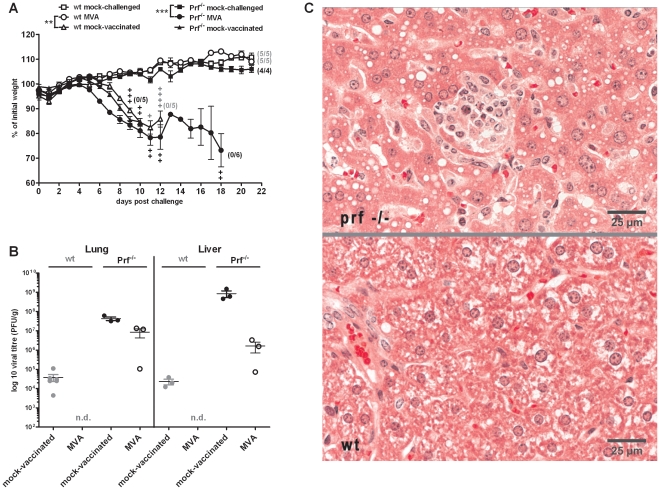
Protective capacity of vaccination is lost in absence of the cytolytic effector molecule perforin. (A) C57BL/6 mice (wt, open symbols) and perforin deficient C57/BL/6 mice (Prf^−/−^, closed symbols) were challenged with 3×LD_50_ ECTV two days after MVA immunization (•), with mock-challenged (▪), and mock-vaccinated (▴) animals as controls. In all experiments weight loss of individual mice was monitored daily (n = 4 to 6 per group). +indicate the individual time of death. Error bars indicate SEMs, and the numbers of surviving/total animals are given in parentheses. Data are representative of two similar experiments. Statistical significance of differences between groups is indicated by * for p-value<0.05, ** for p-value<0.01 and *** for p-value<0.001. (B) At the timepoint of death (day 9 p.i. for Prf^−/−^ mock-vaccinated, day 12 p.i. for Prf^−/−^ MVA and wt mock-vaccinated) or at the end of the experiments (day 21 p.i. for wt MVA) lung and liver were removed, homogenized, and the amount of virus was determined by plaque assay (n = 2 to 3 animals per group). Error bars indicate SEMs and data are representative of at least two independent experiments. n.d.: not detectable. (C) Histopathologic examination of liver tissue from MVA vaccinated and ECTV challenged Prf^−/−^ (upper panel) or wt mice (lower panel) at day 12 p.i.. Tissues were stained with hematoxilin and eosin (HE) and evaluated by light microscopy. Micrographs show representative areas of liver tissue. A typical focus of necrosis and inflammation in the liver of the Prf^−/−^ mouse is visible in the center of the upper micrograph.

T cells seemed to play a dominant role in rapid protection mediated by MVA. CD4+ and CD8+ T cells exert different effector functions to control infections. CD4+ T cells primarily activate other immune cells like B cells and macrophages through expression of cytokines [57]–[59] and they are also recognized as being crucially involved in the activation of antigen-specific CD8^+^ T cells [60]. In contrast, CD8+ T cells can directly kill infected cells which is mediated by the release of cytotoxic granules containing notably perforin and granzymes. Furthermore, studies showed that cell-mediated cytotoxicity and especially perforin is important for recovery from ECTV infection [61], [62]. To analyze the role of perforin mediated cellular cytotoxicity in rapid protection, we vaccinated perforin deficient mice (Prf^−/−^) i.n. with 10^8^ PFU MVA two days before a lethal challenge infection with ECTV. In contrast to wt mice, Prf^−/−^ mice were not protected, developed severe disease and all mice succumbed to ECTV infection until day 18 post infection (Figure 7A). Correspondingly, we detected high levels of virus in lung and liver of MVA vaccinated Prf^−/−^ mice at the time point of death, while vaccinated wt mice had cleared the virus at the end of the experiment (21 dpi) (Figure 7B). Nevertheless, livers of MVA immunized Prf^−/−^ mice contained reduced virus loads in comparison to mock vaccinated Prf^−/−^ controls. This observation appeared to correlate with the somewhat prolonged course of disease in vaccinated animals (not statistically significant) and might be the consequence of innate or humoral adaptive immune responses including the possible contribution of type I and/or type II interferons. Histopathologic examination revealed multiple randomly located foci of necrosis and inflammation in the liver of vaccinated and ECTV challenged Prf^−/−^ mice (Figure 7C, upper panel). These lesions were characterized by hepatocytic necrosis and infiltration by macrophages and lymphocytes. However, MVA immunized wt mice had no necrotic and inflammatory lesions in liver tissues (Figure 7C, lower panel). Thus, the availability of the cytotoxic effector protein perforin was essential to maintain the protective capacity of MVA immunization suggesting the induction of T cell mediated cytotoxicity as key mechanism of protective immunity.

### T cell-dependent rapid protection after conventional smallpox vaccination

**Figure 8:**
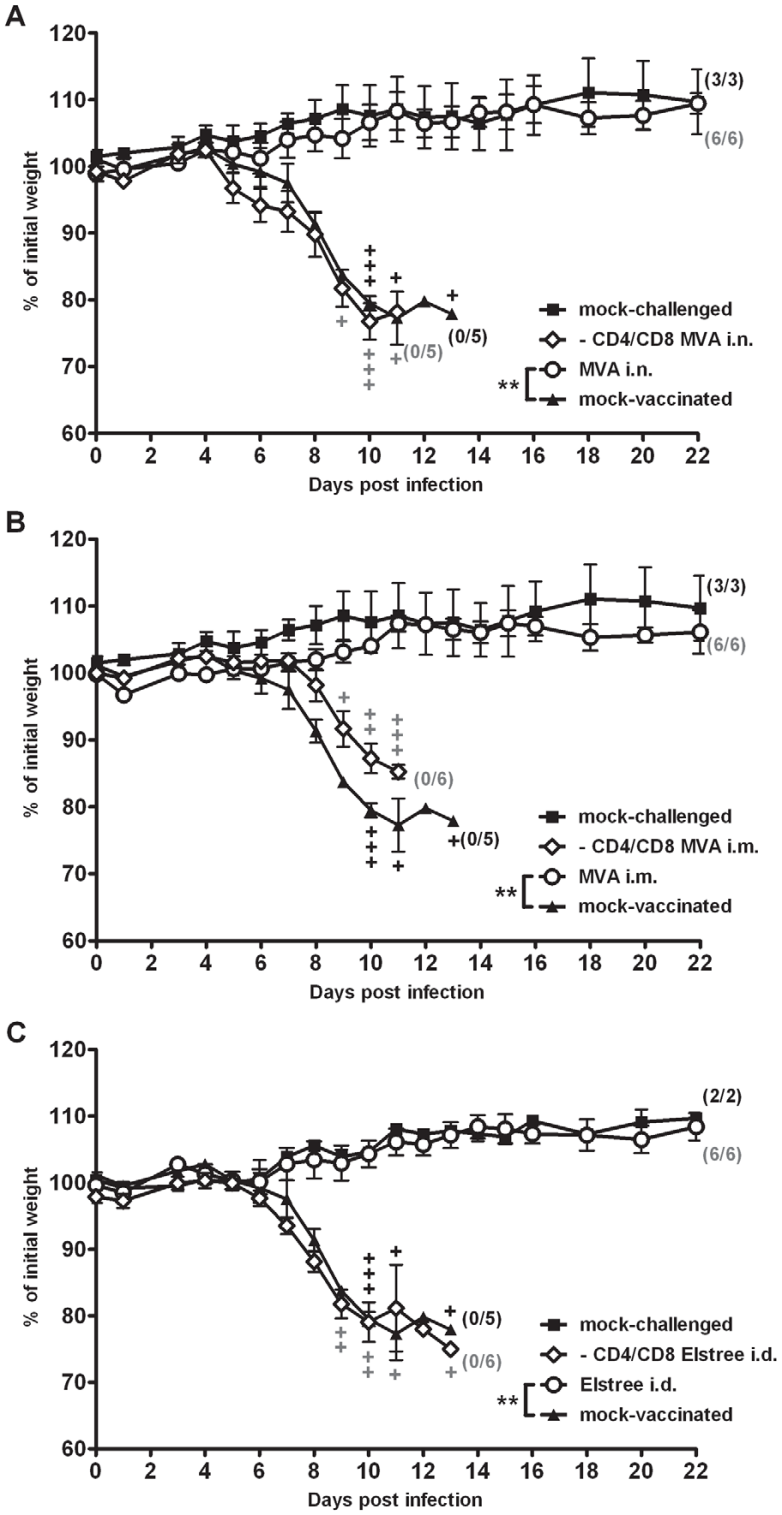
T cell responses are generally required for rapid protection by immunizations with vaccinia virus. C57BL/6 mice (○) or C57BL/6 mice depleted of CD4 and CD8 T cells (◊) were (A) vaccinated with 10^8^ PFU MVA intranasally (i.n.), or (B) intramuscularly (i.m.), or (C) by scarification (i.d.) with 10^6^ PFU VACV Elstree (i.d.) at two days before the 3×LD_50_ ECTV challenge infection. Mock-challenged (▪) and mock-vaccinated (▴) mice served as controls. In all experiments weight loss of individual mice was monitored daily (n = 2 to 6 per group). +indicate the individual time of death. Error bars indicate SEMs, and the numbers of surviving/total animals are given in parentheses. Data are representative of two similar experiments. Statistical significance of differences between groups is indicated by * for p-value<0.05, ** for p-value<0.01 and *** for p-value<0.001.

Intranasal immunizations with VACV are being increasingly investigated as a means to induce immunity associated with the respiratory tract or mucosal tissues. Such applications might be useful in swift mass vaccinations to help overcome major challenges in public health interventions. However, almost all immunizations with VACV in humans, e.g. with the widely used smallpox vaccine strains Lister/Elstree and New York City Board of Health, involved intradermal (i.d.) application through scarification [11]. Clinical uses of MVA-based vaccines routinely choose intramuscular (i.m.) or subcutaneous applications. To elucidate the role of T cells in the rapid protective capacity induced by more conventional vaccination, we again depleted C57BL/6 mice of CD4+ and CD8+ T cells by specific antibodies at the time point of immunization (Figure S6B,C). The rapid protective capacity of i.n. MVA immunization was compared with i.m. MVA immunization using 10^8^ PFU (Figure 8A,B), as well as with i.d. vaccination with 10^6^ PFU of VACV strain Elstree (Figure 8C). Previous studies had confirmed the protective capacity of this lower-dose immunization using the fully replication competent VACV Elstree [41]. As expected, control C57BL/6 wt mice were fully protected by vaccination two days prior to the lethal respiratory challenge infection with ECTV (p = 0.006 for MVA i.n.; p = 0.0011 for MVA i.m.; p = 0.005 for VACV Elstree). In sharp contrast, all vaccinated, T cell-depleted animals succumbed to ECTV infection irrespective of vaccination by MVA or VACV Elstree strains or the different routes of immunization. Additionally, the morbidity profiles of T cell-depleted, vaccinated animals were comparable to mock-vaccinated controls (p = 1 for MVA i.n.; p = 0.3489 for MVA i.m.; p = 1 for VACV Elstree i.d.). Furthermore, the protective capacity of MVA vaccination was lost when i.m. inoculating heat-inactivated MVA doses (corresponding to 10^8^ PFU) (Figure S9) suggesting the need to immunize live MVA vaccine. These data clearly suggest an essential general requirement for both CD8+ and CD4+ T cells in rapidly protective immunization against fatal mousepox.

## Discussion

Vaccination is still the most successful approach to prevent viral diseases. The recent threats of suddenly emerging severe infectious diseases, e.g. caused by severe acute respiratory syndrome coronavirus, West Nile virus, or avian influenza virus, demonstrate the need for new vaccines ready to use in an immediate public health response. Previous studies in animal models for preventing fatal orthopoxvirus disease had shown that immunizations with MVA or conventional VACV could provide protection in a time window close to lethal infection [39], [41], [43]. The purpose of the present study was to determine the immunological mechanisms mediating this rapid protective capacity of MVA vaccination in an orthopoxvirus infection model. Previous studies in the mousepox model had shown that pre- and post-exposure protection can be achieved, and suggested that the induction of adaptive immune responses was essential [41], [42]. Post-exposure immunizations, in particular when given at later times (e.g. 2 days post ECTV infection), can not prevent the onset of severe mousepox disease [41]. However, this feature clearly hampers the definition of immune correlates for MVA vaccine mediated rapid protection (our unpublished data) [63]. In contrast, MVA vaccination two days prior to lethal ECTV challenge allows for solid protection also against the onset of morbidity. Therefore, we chose the pre-exposure immunization model for this study.

The present work was carried out in C57BL/6 mice deficient in various components of the innate or adaptive immune system, exposed to a lethal respiratory infection with ECTV administered two days after immunization. Immunization and challenge of normal C57BL/6 mice served as controls to determine the degree of protection, as monitored by disease symptoms, body weight loss and survival. Moreover, intranasal vaccination of fully immune competent mice allowed us to assess the quality and kinetics of immune responses elicited at the primary site of immunization. Previous studies had suggested that VACV and MVA are recognized via multiple host-sensing pathways, including TLRs, RLRs and NOD-like receptors (NLRs) [44], [64]. Furthermore, MVA infection induces pro-inflammatory cytokines such as TNF-α [44], [65], type I interferons [14],[47],[66] and chemokines like CCL2 that attract leucocytes to the site of inoculation [46]. At 24 to 48 hours after i.n. MVA inoculation we found prominent infiltrations of immune cells in the lungs of immunized mice. We also detected significantly increased amounts of the pro-inflammatory cytokines IL-6 and IFN-α in BAL fluids, which correlate with efficient activation of innate responses in the respiratory tract.

Previous analysis of systemic adaptive immune responses had shown that MVA vaccination elicits strong CD8+ and CD4+ T cell responses with a T-helper type 1 (Th1)-dominant profile [25], [30], [67], [68] as well as orthopoxvirus-specific antibodies [25], [68], [69]. Here we demonstrate that antigen-specific immune responses can be detected in the respiratory tract at early times after immunization. Of note, we found two to three-times higher numbers of IFN-y producing VACV (B8R_20–27_) specific CD8+ T cells in lungs than in spleens. This supports the hypothesis that early local priming of antigen-specific T cells may occur, which is possibly associated with MVA-induced formation of bronchus-associated lymphoid tissue [45]. The rapid expansion of virus-specific CD8+ T cells is further supported by the recent finding of Chaudhri and coworkers that antigen-specific T cell receptors can be transferred and shared among CD8+ T cells to enhance the anti-viral response upon orthopoxvirus infection [70].

Previous work in IFNAR^−/−^ mice had indicated a partial influence of type I IFN in rapid protection mediated by MVA vaccination [41]. Here we show that the protective capacity of MVA immunization was fully maintained in MyD88/Trif^−/−^ and IPS^−/−^ mice that are deficient in TLR and RLR signaling pathways controlling the expression of type I IFN. Nevertheless, it cannot be excluded that other innate recognition pathways are involved in mediating a type I IFN response to MVA. Thus it seems likely that due to versatile recognition of MVA, signaling pathways can compensate for each other to provide the innate responses essential for developing protective adaptive immunity. In particular, further study of the possible contribution of type I IFN in rapidly protective MVA immunization seems promising because concomitant type I interferon receptor triggering on T cells and DC has been recently shown to allow for optimal expansion of MVA induced CD8 T cell responses [71].

NK cells are part of the cellular innate immune response and recent work established their key role in host specific control of a primary ECTV infection [51], [52]. Interestingly, we found here that depletion of NK cells did not influence the rapid protective capacity of MVA vaccination. This observation already indicated differences in the modes of immune defense when comparing rapidly protective primary immunization, protection against secondary infection, and overcoming a primary ECTV infection. This latter scenario is well characterized in C57BL/6 mice that resist foot-pad infections known to be lethal in more susceptible mouse strains. This primary resistance was shown to mainly depend on the presence of NK cells and T cells. Nevertheless, B cells are essential for complete virus clearance and recovery [50], [52], [72], [73]. In contrast, protective immunity against secondary infections, elicited by primary infection or conventional vaccination, is dominated by antibody responses [23], [27], [40], [74]–[76]. Recovery from secondary ECTV infection was demonstrated to rely on a more rapid recall antibody response than in primary infection, whereas secondary recall CTL responses were not altered compared to primary CTL responses [77]. Nevertheless, memory CD8+ T cells are known to prevent viral spread by killing viral targets in the draining lymph nodes and thus are also important in controlling secondary ECTV infections [78]. Thus, the antibody memory response seems to be mandatory for long-term protective immunity against orthopoxvirus infections especially in controlling virus persistence while T cell responses might be more important to prevent viral spread.

Previous immunization experiments in T and B cell deficient RAG-1^−/−^ mice had already indicated that adaptive responses are indispensible to achieving rapidly protective immunity [41], [42]. We also suspected a key role of humoral immunity in rapidly protective immunization, since MVA can induce antibody responses much faster than conventional VACV [39], [41]. Moreover vaccinia immune globulin is effective in post-exposure treatment of lethal orthopoxvirus infections [28], [53], [79]. However surprisingly, we found that vaccinated B cell-deficient mice were still fully protected. MVA immunization prevented the onset of any detectable disease in B cell deficient animals for at least four weeks following respiratory challenge infection. This is remarkable because in this intranasal infection model (at low dosage of 200 PFU ECTV) normal C57BL/6 mice (not vaccinated) suffer from severe systemic mousepox and succumb within 10 to 14 days after challenge [80]. It is worth noting, however, that ECTV can persist for several months without any signs of disease in naïve C57BL/6 mice following footpad inoculation and, in infected B cell deficient animals, the onset of symptoms must not occur until very late in infection [72], [73]. Yet, on the contrary, depletion of CD4+ or/and CD8+ T cells in C57BL/6 mice completely abrogated the protective capacity of immunization against the respiratory ECTV challenge. Moreover, the need for T cell-mediated immunity was underlined by the transfer of naïve CD3+ T cells into RAG-1^−/−^ mice, which supported protective vaccination of these immunocompromised animals against lethal ECTV challenge. Furthermore, depletion of CD4+ T cells was sufficient to inhibit the protective effect of MVA immunization, although we observed clearly delayed onset of morbidity. This observation may be best explained by an essential role of T helper cells in mediating efficient clearance of virus by CD8+ T cell activity. Importantly, this possibility is clearly supported by our demonstration that depletion of CD4+ T cells significantly reduced the *in vivo* expansion of endogenous VACV-specific CD8+ T cells. Similarly, CD4+ T cells have been found essential for maintaining a robust or protective cytotoxic T cell memory response upon vaccination with recombinant VACV expressing lymphocytic choriomeningitis virus glycoprotein, or upon infection of mice with *Listeria monocytogenes* bacteria [81], [82], [83] The possibility that clearance of ECTV cannot be accomplished because of the absence of T helper cell dependent antibody responses appears unlikely in the view of the fact that CD4-depleted animals still mounted substantial levels of VACV-specific antibodies. Moreover, we clearly demonstrated the essential need for the direct cytotoxic effector function of CD8+ T cells to mediate rapid protection as the absence of perforin completely abrogated the protective capacity of immunization. Nonetheless, we observed reduced levels of ECTV also in the livers of vaccinated Prf−/− mice indicating that MVA induced innate responses might have modulated the course of infection. This hypothesis is in agreement with previous findings of an early enhanced production of chemokines and cytokines after *in vivo* inoculation of MVA but not other strains of VACV [46], [84].

Importantly, we confirmed the necessity of T cells in rapid protection not only with MVA immunizations via the intramuscular route, but also conventional scarification using VACV strain Elstree vaccine. These data suggests that the need for T cell-mediated immunity is independent of the vaccination route or vaccine strain used. Moreover, this indicates a general requirement of T cells for rapidly protective immunizations against orthopoxvirus infections, and maybe also against other infectious diseases that necessarily fit a scenario of emergency vaccination. In addition, we present here an outstanding experimental model for immunizations with a live viral vaccine suitable for use in humans, where protective vaccination strictly depends on T cell responses. Future work with this model should help in the development of new vaccines eliciting more effective T cell mediated immunity.

## Materials and Methods

### Ethics statement

This study was carried out in strict accordance with German regulations for animal experimentation (German Animal Welfare Act). All experimentations were approved by the Government of the State of Hesse (Paul-Ehrlich-Institut, Permit Numbers 107/65, 107/67, 107/82), the Government of Upper Bavaria (University of Munich LMU, Permit Number 59.10) and the Niedersächsische Landesamt für Verbraucherschutz und Lebensmittelsicherheit (LAVES). All intranasal inoculations were performed under ketamine/xylazine anesthesia, and all efforts were made to minimize suffering of infected animals.

### Cells and viruses

Monkey BS-C-1 (ATCC CCL-26), murine NIH3T3 (ATCC CRL-1658), human monocytic THP-1 (German Collection of Cell Culture DSMZ, Braunschweig, Germany) and chicken embryo fibroblast (CEF) cells were used and routinely maintained as previously described [25]. [46]. Plaque purified Ectromelia virus (ECTV) strain Moscow (ATCC VR-1374, kindly provided by Mark L. Buller, St. Louis University School of Medicine, St. Louis, Missouri, USA) was propagated on BS-C-1 cells. Modified vaccinia virus Ankara (MVA) (clonal isolate F6) [16], [22] was propagated on CEF cells. Viral titers were determined by plaque assay and titrated in plaque forming units (PFU) as previously described [25], [85].

### Mice

Female C57BL/6N mice (6–10 weeks old) were purchased from Charles River Laboratories (Sulzfeld, Germany). C57BL/6J-Igh-6^tm1Cgn^ mice (μMT, immunoglobulin heavy chain 6 deficient [heavy chain of IgM]) [54] and C57BL/6-Prf1^tm1Sdz^/J mice (Prf^−/−^, perforin deficient) [81]. were purchased from The Jackson Laboratory. C57BL/6J-Rag1^tm1Mom^ mice (RAG-1^−/−^, mice homozygous for the *Rag1^tm1Mom^* mutation produce no mature T cells or B cells) [86], C57BL/6 MyD88^−/−^ TRIF^−/−^ mice [47], [87], C57BL/6 IPS-1^−/−^ mice [88] and C57BL/6-Igh-Jtm1Cgn/J mice (JHT, mice homozygous for the *Igh-J^tm1Cgn^* targeted mutation fail to produce functional B cells) [55], [89] were bred under specific pathogen-free conditions at the central animal facility of the Paul-Ehrlich Institute. For experimental work, mice were housed in an ISOcage unit (Tecniplast, Germany) and had free access to food and water. All animal experiments were handled in compliance with the German regulations for animal experimentation (Animal Welfare Act).

### Immunization experiments

Intradermal (i.d.) vaccination was performed by tail scarification as described elsewhere [41]. Briefly, 10 µl of virus suspension containing 10^6^ PFU VACV Elstree was deposited on the mouse skin at the tail base. The skin was then scratched through the droplet with the tip of a 26-gauge needle (Braun, Melsungen, Germany) to allow virus uptake. For intramuscular (i.m.) vaccination, 50 µl of virus suspension containing 10^8^ PFU of MVA or PBS as a mock control were injected into the right hind leg. Intranasal (i.n.) immunization was performed as described elsewhere [24], [43]. Briefly, mice were anesthetized by intraperitoneal (i.p.) injection with 1 mg ketamine and 0.04 mg xylazine per 10 g body weight and instilled i.n. with 30 µl of virus suspension containing 10^8^ PFU of MVA. In all experiments inoculations of corresponding amounts of PBS were used as controls (mock vaccine).

### Challenge experiments

Mice were anesthetized by intraperitoneal (i.p.) injection with 1 mg ketamine and 0.04 mg xylazine per 10 g body weight and infected by i.n. inoculation with 200 PFU (∼3×LD_50_) ECTV virus suspension as described previously [43]. Signs of illness, weight loss and survival were monitored daily for at least three weeks.

### Depletion of specific subsets of immune cells

Mice were depleted of CD4+ T cells, CD8+ T cells, or NK cells by i.p. administration of mouse monoclonal antibodies purchased from Harlan Bioproducts, Indianapolis, USA. CD4+ T cells were depleted by applying 500 µg of anti-CD4 clone GK1.5 antibody [90] on days −8, −6, −3, −2, and −1 prior to immunization on day 0. CD8+ T cell depletion was performed by administration of 100 µg anti-CD8 clone 2.43 antibody [91] on days −2 and −1 prior to immunization on day 0. Depletion of both CD4+ and CD8+ T cells was achieved by combining the two described applications of GK1.5 and 2.43 antibodies. NK cells were depleted with an anti-NK1.1 clone PK136 antibody [92] applying 300 µg of antibody on days −2 and −1 prior to immunization on day 0. Successful depletion of immune cells was confirmed by flow cytometric analysis of spleen cells from antibody treated animals on days 0 and 7 post immunization.

### Adoptive T cell transfer

Spleens were isolated from euthanized C57BL/6 mice in pre-warmed RPMI medium enriched with 10% fetal calf serum. Single cell suspensions were obtained by passing cells through a nylon mesh (Nybolt PA-150/38, Franz Eckert GmBH, Germany) and erythrocytes were lysed by treatment with Red blood cell lysis buffer (Sigma Aldrich, Taufkirchen, Germany). Subsequently, cells were washed with medium and passed through a 70 µm filter (Filcon, BD Biosciences, Heidelberg, Germany) and again washed with medium. For isolation of untouched T cells the spleen cell suspension was magnetically labeled using the Pan T Cell Isolation Kit (Miltenyi, Bergisch Gladbach, Germany) and isolated using an autoMacs™ separator according to the manufacturer's protocol. Purity of the isolated T cells was confirmed by flow cytometry. Rag-1^−/−^ mice were injected intravenously with 2×10^7^ CD3+ cells per mouse two days before immunization and the engraftment was confirmed by FACS analysis of blood samples on days 4, 9, 11, 15, 21 and 37 after administration.

### Histopathology

Lungs from sacrificed mice were fixed by instilling formaldehyde solution (4%, pH 7.2) through the trachea. Inflated lungs and livers were removed and fixed in phosphate buffered formalin. Tissues were embedded in paraffin and sections (4 µm) were stained with hematoxylin and eosin before being evaluated by light microscopy.

### Bronchoalveolar lavages (BAL)

Lungs from sacrificed mice were inflated three times with 0.7 ml PBS using a stainless steel buttoned cannula (ACUFIRM, Ernst Kratz KG Nadelfabrik, Germany 1428 LL). Pooled fluids (n = 3) were collected and cells were harvested by low-spin centrifugation for antibody staining and FACS analysis. The cell-free fluids of the first instillation were collected and stored at −80°C for further analysis to detect cytokines and antibodies by ELISA.

### Flow cytometry

Approximately 10^5^ cells were stained in 50 µl PBS supplemented with 3% FCS using monoclonal antibodies obtained from BD Biosciences (Heidelberg, Germany). Monocytes, neutrophils, dendritic cells, NK cells, B cells, and T cells were detected using APC-labeled CD11c, PerCP-Cy5.5-labeled CD11b, PE-Cy7-labeled Gr-1, PE-labeled CD49b, PE-Cy7-labeled NK1.1, APC-Cy7-labeled B220, PerCp-Cy5.5-labeled CD3, PE-labeled CD4 and FITC-labeled CD8 antibodies. To ensure specificity of staining, all staining tests contained an isotype-matched control antibody. Stained cells were fixed with PBS supplemented with 0.5% paraformaldehyde and analyzed with BD LSRII and BD FACSDiva 6.0 software (BD Biosciences, Heidelberg, Germany).

### Analysis of antigen-specific CD8+ T cells by intracellular cytokine staining

Splenocytes or BAL cells from vaccinated C57BL/6 mice were stimulated for 5 h with VACV-specific peptide B8R_20–27_ (TSYKFESV) [48] or control peptide LacZ_876_ (TPHPARIGL) [93] purchased from Thermo Fisher Scientific GmbH (Ulm, Germany) in the presence of GolgiStop™ (BD Biosciences, Heidelberg, Germany). Cells were blocked with anti-CD16/CD32-Fc-Block (BD Biosciences) and surface markers were stained with PacBlue-conjugated anti-CD8 (BD Biosciences) and APC-conjugated anti-CD62L (BD Biosciences, Heidelberg, Germany) in the presence of Fc-Block (BD biosciences, Heidelberg, Germany) and washed twice with PBS containing 3% FCS. Intracellular cytokine staining for IFN-γ production was performed with FITC anti–IFN-γ (BD Biosciences, Heidelberg, Germany) using the Cytofix/Cytoperm kit (BD Biosciences, Heidelberg, Germany) according to the manufacturer's recommendations. Data were acquired in a BD LSRII flow cytometer and analyzed with BD FACSDiva 6.0 software (BD Biosciences, Heidelberg, Germany).

### Measurement of cytokines in BAL fluids

Supernatants of the first BAL instillations were collected and pooled from 3 mice. Detection of different cytokines used undiluted BAL fluids for ELISA in triplicates. Measurement of interleukin-6 (IL-6) used a Quantikine ELISA Kit purchased from R&D Systems (Wiesbaden-Nordenstadt, Germany). ELISA for detecting interferon-α (IFN-α) was purchased from PBL InterferonSource (distributed by tebu-bio GmbH, Offenbach, Germany). Assays were performed according to manufacturer's instructions and repeated at least three times.

### Measurement of vaccinia virus specific antibodies by ELISA

ELISA plates (MaxiSorp 96-well flat-bottom, Nunc, Wiesbaden, Germany) were coated with sucrose gradient-purified MVA (at a protein concentration of 1 µg/ml) for 3 h at 37°C and overnight at 4°C. The plates were blocked with PBS containing 0.05% Tween 20 and 10% fetal calf serum for 60 min at 37°C. BAL fluids were incubated for 60 min at 37°C, washed five times with PBS, and then incubated for 30 min with a goat anti-mouse IgG conjugated to horseradish peroxidase (HRP) (Kirkegaard & Perry Laboratories, Gaithersburg USA) (diluted 1∶2000 in PBS). After five washes, the plates were incubated with OPD substrate (Sigma, Taufkirchen, Germany) at room temperature for 5–10 min. The optical density was measured immediately after addition of stop solution (0.5 M sulfuric acid) at a wavelength of 490 nm [94].

### Reverse transcriptase-PCR (RT-PCR)

RNA isolation and amplification of human GAPDH cDNA were performed as described [95] using 26 amplification cycles. Similarly, amplification of murine GAPDH cDNA was performed with the sense primer 5′-GAC AAC TCA CTC AAG ATT GTC AG-3′ and the antisense primer 5′-GTA GCC GTA TTC ATT GTC ATA CC-3′, resulting in a product size of 540 bp. Amplification of VACV B8R gene (GenBank accession no. AY603355) was undertaken using the sense primer 5′-TAA AAA TTA TGG CAT CAA GAC G-3′ and the antisense primer 5′-ACA TCT TCT TTG GAT CTA ATT GC-3′, resulting in a product size of 495 bp for MVA, and 540 bp for VACV strain Elstree. The MVA E3L gene orthologue has been amplified using the sense primer 5′-TTA CTA GGC CCC ACT GAT TC-3′ and the antisense primer 5′-GTT CTG ACG CAG AGA TTG TG-3′, resulting in a product size of 406 bp. Primer pairs were designed using Primer3 software [96]. All oligonucleotides were synthesized by Eurofins MWG Operon GmbH (Ebersberg, Germany). PCR products were run on a 1.5% agarose gel and stained with GelRed purchased from MoBiTec (Göttingen, Germany). Gel pictures acquired by a CCD camera were analyzed using the Photo-Capt 12.4 software (Vilber Lourmat, Eberhardzell, Germany).

### Statistical analysis

Statistical comparison of different groups of mice was performed as means of the area under the weight curve (AUC) in percent of individual weight at baseline. The AUC was additionally weighted with the length of the observation period (usually day of challenge (day 0) until day 22, or the day the animal died). The differences between vaccination groups were analyzed with a one-factorial analysis of variance model. For multiple comparisons p-values were adjusted with the Bonferroni method. The statistical evaluation was performed with SAS/STAT software, version 9.2, SAS System for Windows. For statistical significant results the following convention was used: * – p-value<0.05, ** – p-value<0.01 and *** – p-value<0.001.

## Supporting Information

Figure S1 Intranasal immunization with MVA induces peribronchiolar and perivascular infiltrate of leukocytes in the lung. Histopathological examination of lungs from C57BL/6 mice after intranasal (i.n.) inoculation with mock vaccine (top panel) or 10^8^ PFU MVA (lower panel). At 48 hours after inoculation inflated lungs were fixed with 4% formalin and embedded in paraffin. Sections were stained with hematoxilin and eosin (HE). Overview images demonstrate the extent of the infiltrate developing after MVA inoculation.
(TIF)

Figure S2 Intranasal immunization with MVA induces peribronchiolar and perivascular infiltrate of leukocytes in the lung. Histopathological examination of lungs from C57BL/6 mice after intranasal (i.n.) inoculation with mock vaccine (top panel) or 10^8^ PFU MVA (lower panel). At 48 hours after inoculation inflated lungs were fixed with 4% formalin and embedded in paraffin. Sections were stained with hematoxilin and eosin (HE). Images at higher magnification show the presence of neutrophils and macrophages.
(TIF)

Figure S3 Rapid infiltration of innate immune cells after i. n. immunization with MVA. The bronchoalveolar lavage (BAL) data are representative of two independent experiments. Mice (n = 3) were immunized with either mock vaccine (PBS) (○) or MVA (1×10^8^ PFU) (•) and BAL performed at the indicated time points. (A) Pooled BAL cells were analyzed by FACS to detect monocytes (CD11b+), (B) dendritic cells (DC) (CD11b+ CD11c+), (C) neutrophils (CD11b+ Gr-1^high^) and (D) natural killer (NK) cells (CD3− NK1.1+). Total cell numbers are shown.
(TIF)

Figure S4 B8R gene products are expressed at equal levels by MVA and conventional VACV strain Elstree/Lister. (A) Schematic representation of the B8R coding sequences (CDS) in the genomes of VACV Elstree/Lister (CDS 257) and MVA (CDS 176). The gene products are depicted by grey arrows and the sites of truncations within the MVA B8 protein are shown by white boxes. A hatched box indicates the position of the conserved peptide epitope B8R_20–27_. (B) MVA or VACV Elstree/Lister specific B8R gene products were analyzed by RT-PCR. NIH 3T3 cells were infected with virus at an MOI of 20 and total RNA was prepared at 2 and 4 hours post infection (h p.i.). RNA from mock infected cells and GAPDH specific RT-PCR served as controls.
(TIF)

Figure S5 Rapid activation of innate immune responses after i. n. immunization with MVA. The bronchoalveolar lavage (BAL) data are representative of two independent experiments. Mice (n = 3) were immunized with either mock vaccine (PBS) (□) or MVA (1×10^8^ PFU) (▪) and BAL performed at the indicated time points. (A) Pooled BAL fluids were analyzed for interferon-α (IFN-α), and (B) interleukin-6 (IL-6) by ELISA.
(TIF)

Figure S6 Efficient depletion of NK cells, CD8+, and CD4+ T cells at the time point of immunization. Mice were depleted of CD4+ T cells, CD8+ T cells, and NK cells by intraperitoneal (i.p.) administration of mouse monoclonal antibodies. Spleen cells were stained for different cell surface markers and analyzed by FACS. The percentage of (A) NK cells (CD3−, NK1.1+, CD49b+), (B) CD8+ T cells (CD3+, CD8+) and (C) CD4+ T cells (CD3+, CD4+) from antibody treated mice was compared to untreated mice.
(TIF)

Figure S7 Protective immunization is independent of the presence of B cells. B cell-deficient JHT mice were i.n. immunized with MVA (○) two days before 3×LD_50_ ECTV challenge. Mock-challenged (▪) and mock-vaccinated (▴) mice served as controls. In all experiments weight loss of individual mice was monitored daily (n = 2 to 3 per group). +indicate the individual time of death. Error bars indicate SEMs, and the numbers of surviving/total animals are given in parentheses.
(TIF)

Figure S8 C57BL/6 mice and B cell-deficient JHT mice mount comparable VACV specific T cell responses. At 7 days after MVA immunization spleen cells from individual C57BL/6 (wt) (n = 6) mice or B-cell deficient JHT mice (n = 5) were stimulated with VACV specific peptide B8R_20–27_ and subsequently CD8+ CD62L^low^ T cells were analyzed by intracellular cytokine staining and FACS for gamma interferon expression (IFN-y+).
(TIF)

Figure S9 Heat-inactivated MVA vaccine does not protect from morbidity and mortality following ECTV challenge. (A) C57BL/6 mice were i.m. immunized with MVA (10^8^ PFU) (n = 5) or heat-inactivated MVA (corresponding to 10^8^ PFU) (n = 5) two days before 3×LD_50_ ECTV challenge. Mock-challenged (▪) (n = 3) and mock-vaccinated (▴) (n = 5) mice served as controls. In all experiments weight loss of individual mice was monitored daily (n = 3 to 5 per group). The data shown are representative for two similar experiments. +indicate the individual time of death. Error bars indicate SEMs, and the numbers of surviving/total animals are given in parentheses. Statistical significance of differences between groups is indicated by * for p-value<0.05, ** for p-value<0.01 and *** for p-value<0.001. (B) Confirmation of MVA inactivation. Heat-treatment (60°C for 4 hours) of MVA vaccine preparation prevents activation of viral early gene transcription. Human THP-1 cells were infected with MVA or heat-treated MVA (corresponding to an MOI of 4) and incubated for 6 h at 37°C. Total RNA was isolated from infected and mock-infected cells, and analyzed by RT-PCR using specific oligonucleotide primers for the products of VACV early gene E3L and human GAPDH.
(TIF)
