# Supplementary figures and images for: Critical Role of Perforin-dependent CD8+ T Cell Immunity for Rapid Protective Vaccination in a Murine Model for Human Smallpox
Source: PLoS Pathog. 2012 Mar 1;8(3):e1002557. doi: 10.1371/journal.ppat.1002557 (PMC3291617; doi:10.1371/journal.ppat.1002557)

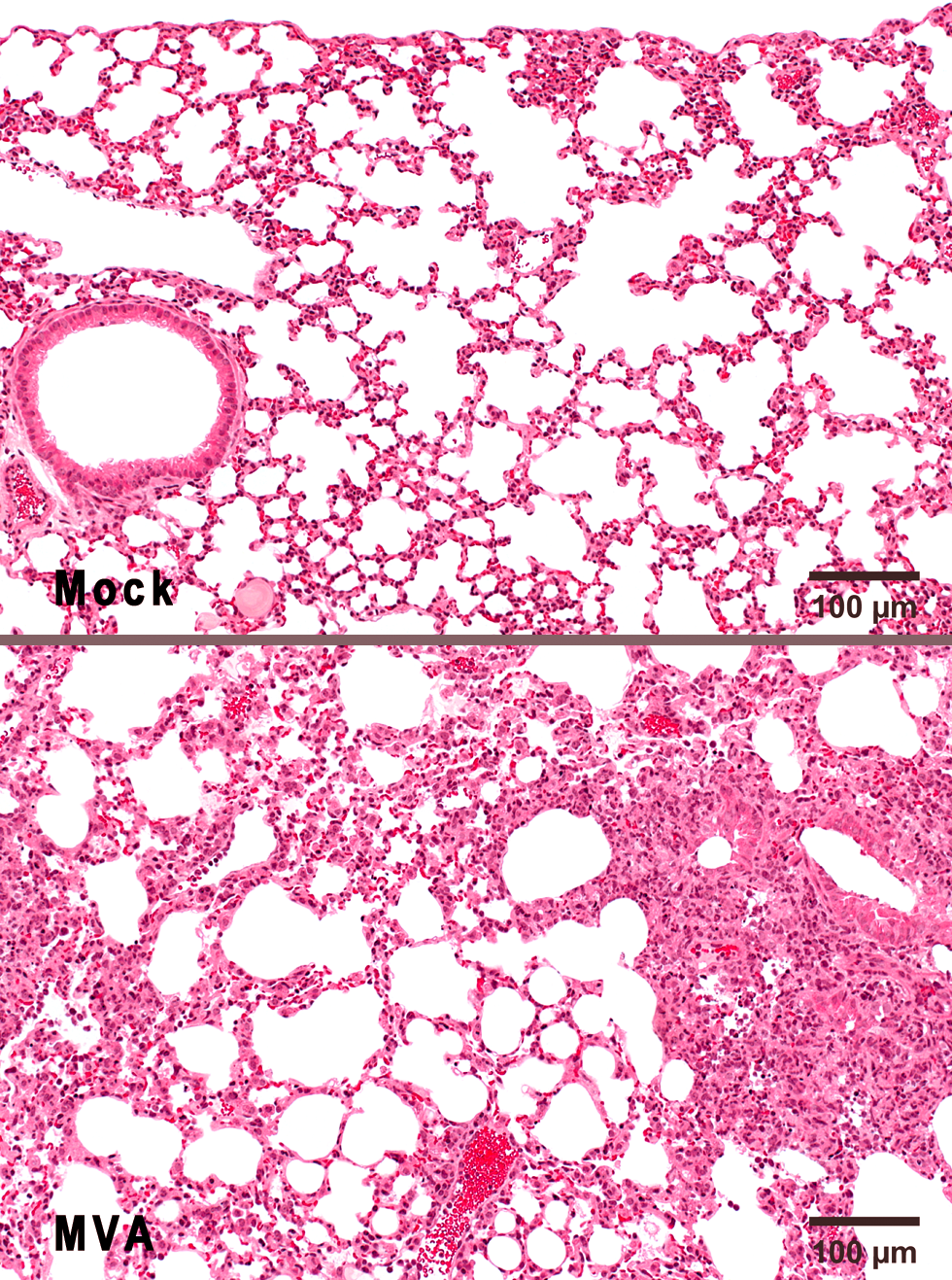

Supplement: Figure S1 — Intranasal immunization with MVA induces peribronchiolar and perivascular infiltrate of leukocytes in the lung. Histopathological examination of lungs from C57BL/6 mice after intranasal (i.n.) inoculation with mock vaccine (top panel) or 108 PFU MVA (lower panel). At 48 hours after inoculation inflated lungs were fixed with 4% formalin and embedded in paraffin. Sections were stained with hematoxilin and eosin (HE). Overview images demonstrate the extent of the infiltrate developing after MVA inoculation. (TIF) [file ppat.1002557.s001.tif]

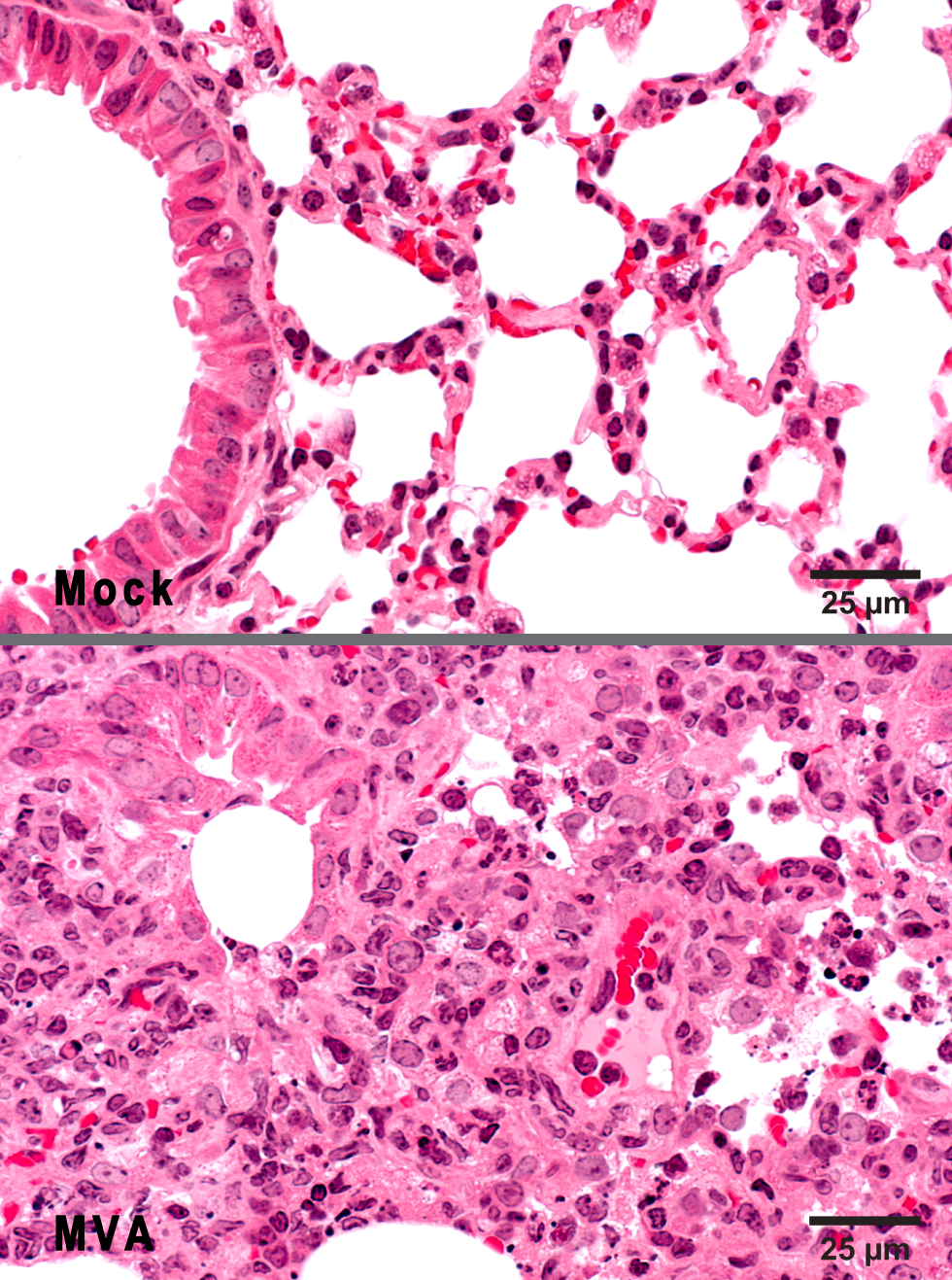

Supplement: Figure S2 — Intranasal immunization with MVA induces peribronchiolar and perivascular infiltrate of leukocytes in the lung. Histopathological examination of lungs from C57BL/6 mice after intranasal (i.n.) inoculation with mock vaccine (top panel) or 108 PFU MVA (lower panel). At 48 hours after inoculation inflated lungs were fixed with 4% formalin and embedded in paraffin. Sections were stained with hematoxilin and eosin (HE). Images at higher magnification show the presence of neutrophils and macrophages. (TIF) [file ppat.1002557.s002.tif]

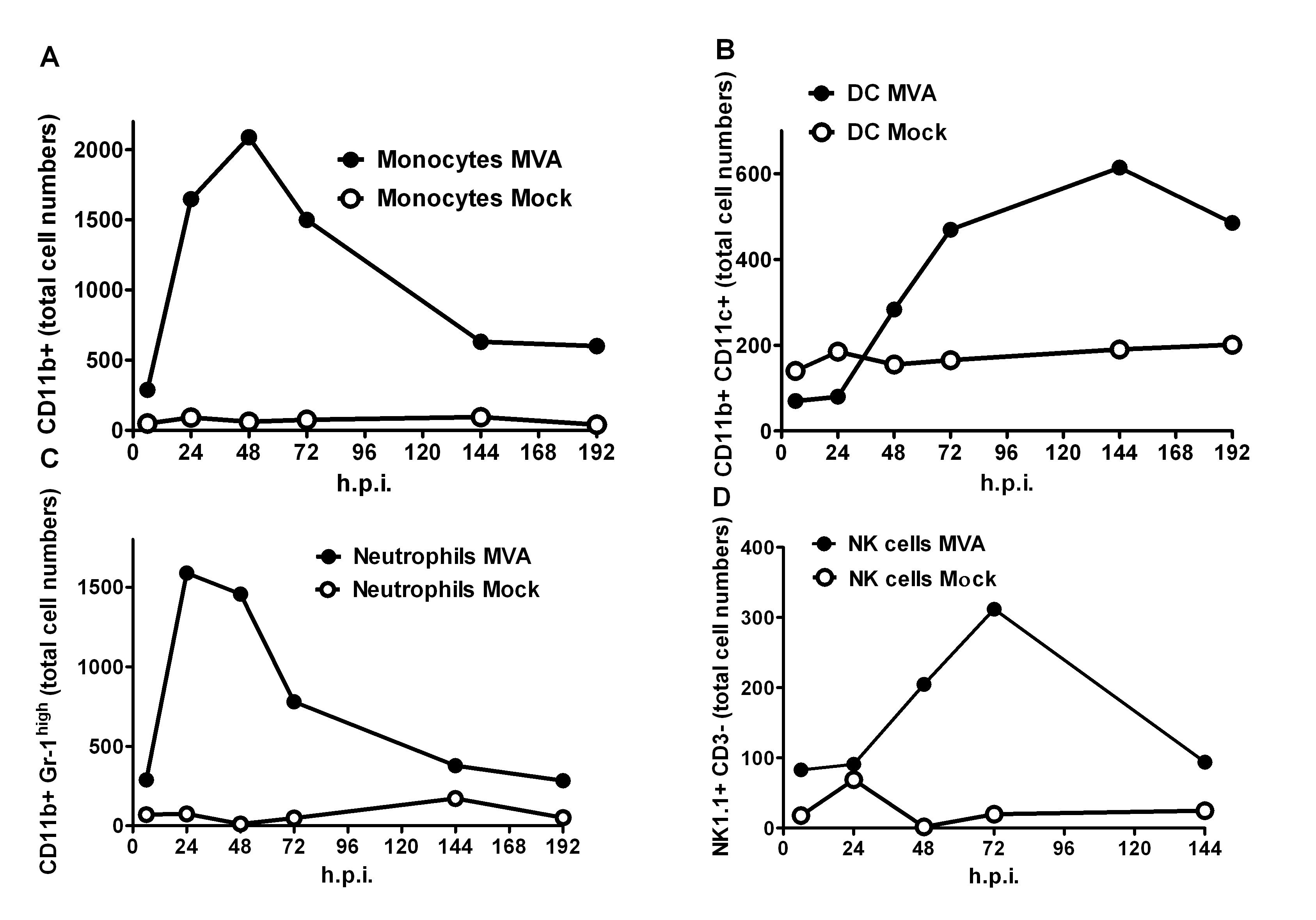

Supplement: Figure S3 — Rapid infiltration of innate immune cells after i. n. immunization with MVA. The bronchoalveolar lavage (BAL) data are representative of two independent experiments. Mice (n = 3) were immunized with either mock vaccine (PBS) (○) or MVA (1×10^8^ PFU) (•) and BAL performed at the indicated time points. (A) Pooled BAL cells were analyzed by FACS to detect monocytes (CD11b+), (B) dendritic cells (DC) (CD11b+ CD11c+), (C) neutrophils (CD11b+ Gr-1high) and (D) natural killer (NK) cells (CD3− NK1.1+). Total cell numbers are shown. (TIF) [file ppat.1002557.s003.tif]

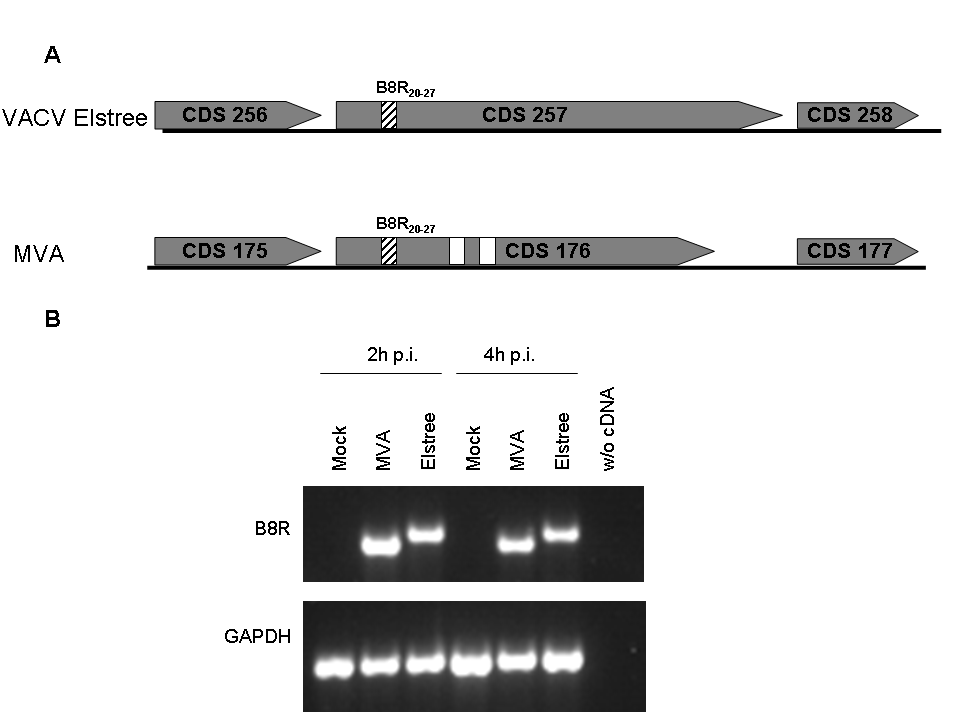

Supplement: Figure S4 — B8R gene products are expressed at equal levels by MVA and conventional VACV strain Elstree/Lister. (A) Schematic representation of the B8R coding sequences (CDS) in the genomes of VACV Elstree/Lister (CDS 257) and MVA (CDS 176). The gene products are depicted by grey arrows and the sites of truncations within the MVA B8 protein are shown by white boxes. A hatched box indicates the position of the conserved peptide epitope B8R20–27. (B) MVA or VACV Elstree/Lister specific B8R gene products were analyzed by RT-PCR. NIH 3T3 cells were infected with virus at an MOI of 20 and total RNA was prepared at 2 and 4 hours post infection (h p.i.). RNA from mock infected cells and GAPDH specific RT-PCR served as controls. (TIF) [file ppat.1002557.s004.tif]

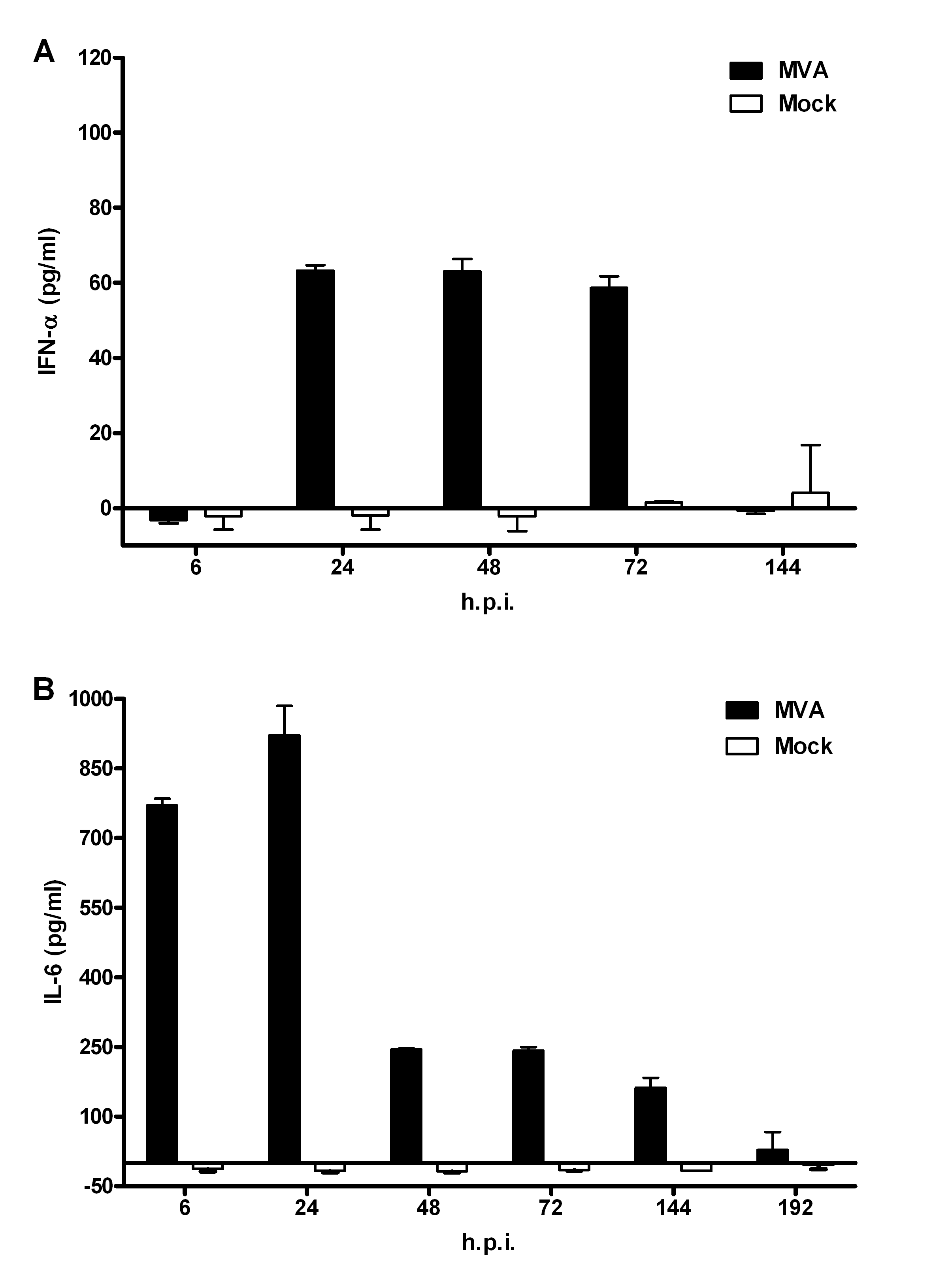

Supplement: Figure S5 — Rapid activation of innate immune responses after i. n. immunization with MVA. The bronchoalveolar lavage (BAL) data are representative of two independent experiments. Mice (n = 3) were immunized with either mock vaccine (PBS) (□) or MVA (1×10^8^ PFU) (▪) and BAL performed at the indicated time points. (A) Pooled BAL fluids were analyzed for interferon-α (IFN-α), and (B) interleukin-6 (IL-6) by ELISA. (TIF) [file ppat.1002557.s005.tif]

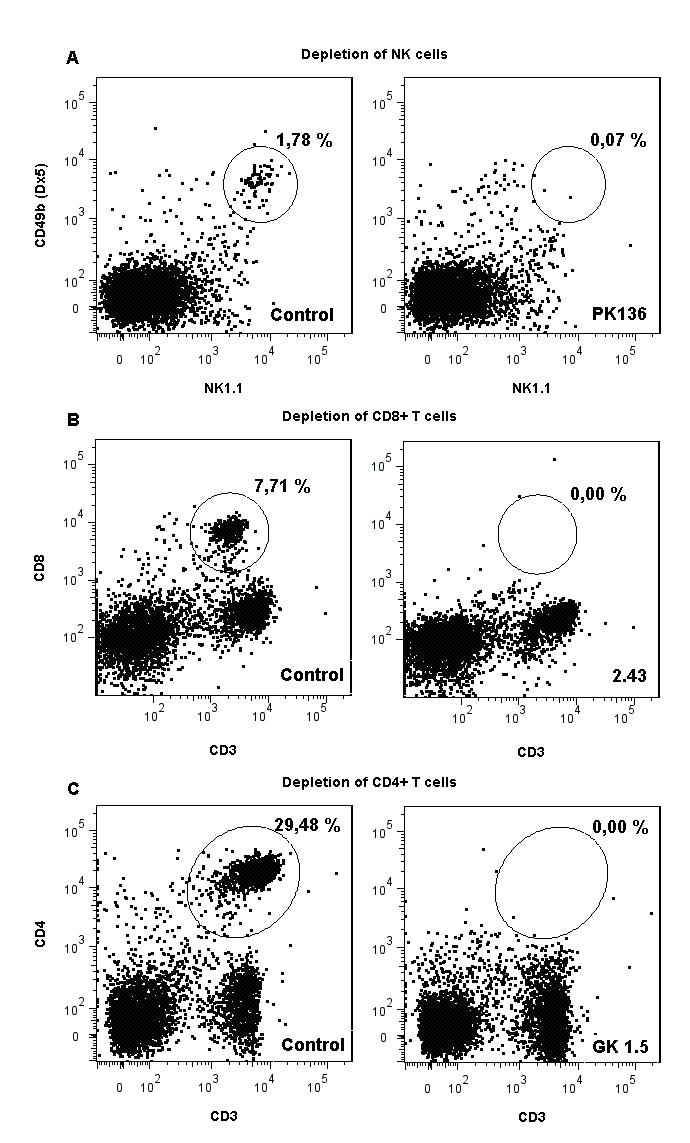

Supplement: Figure S6 — Efficient depletion of NK cells, CD8+, and CD4+ T cells at the time point of immunization. Mice were depleted of CD4+ T cells, CD8+ T cells, and NK cells by intraperitoneal (i.p.) administration of mouse monoclonal antibodies. Spleen cells were stained for different cell surface markers and analyzed by FACS. The percentage of (A) NK cells (CD3−, NK1.1+, CD49b+), (B) CD8+ T cells (CD3+, CD8+) and (C) CD4+ T cells (CD3+, CD4+) from antibody treated mice was compared to untreated mice. (TIF) [file ppat.1002557.s006.tif]

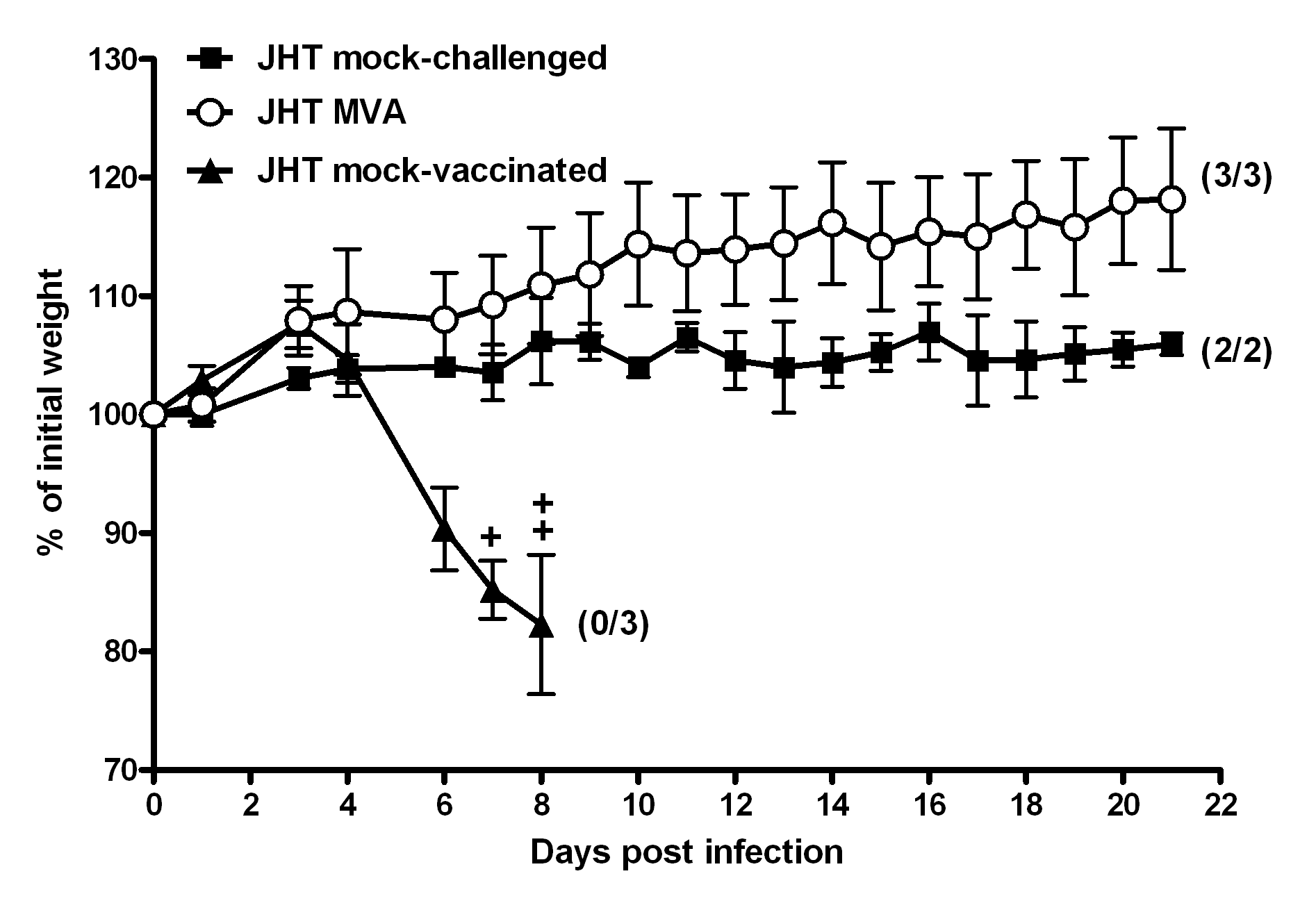

Supplement: Figure S7 — Protective immunization is independent of the presence of B cells. B cell-deficient JHT mice were i.n. immunized with MVA (○) two days before 3×LD50 ECTV challenge. Mock-challenged (▪) and mock-vaccinated (▴) mice served as controls. In all experiments weight loss of individual mice was monitored daily (n = 2 to 3 per group). +indicate the individual time of death. Error bars indicate SEMs, and the numbers of surviving/total animals are given in parentheses. (TIF) [file ppat.1002557.s007.tif]

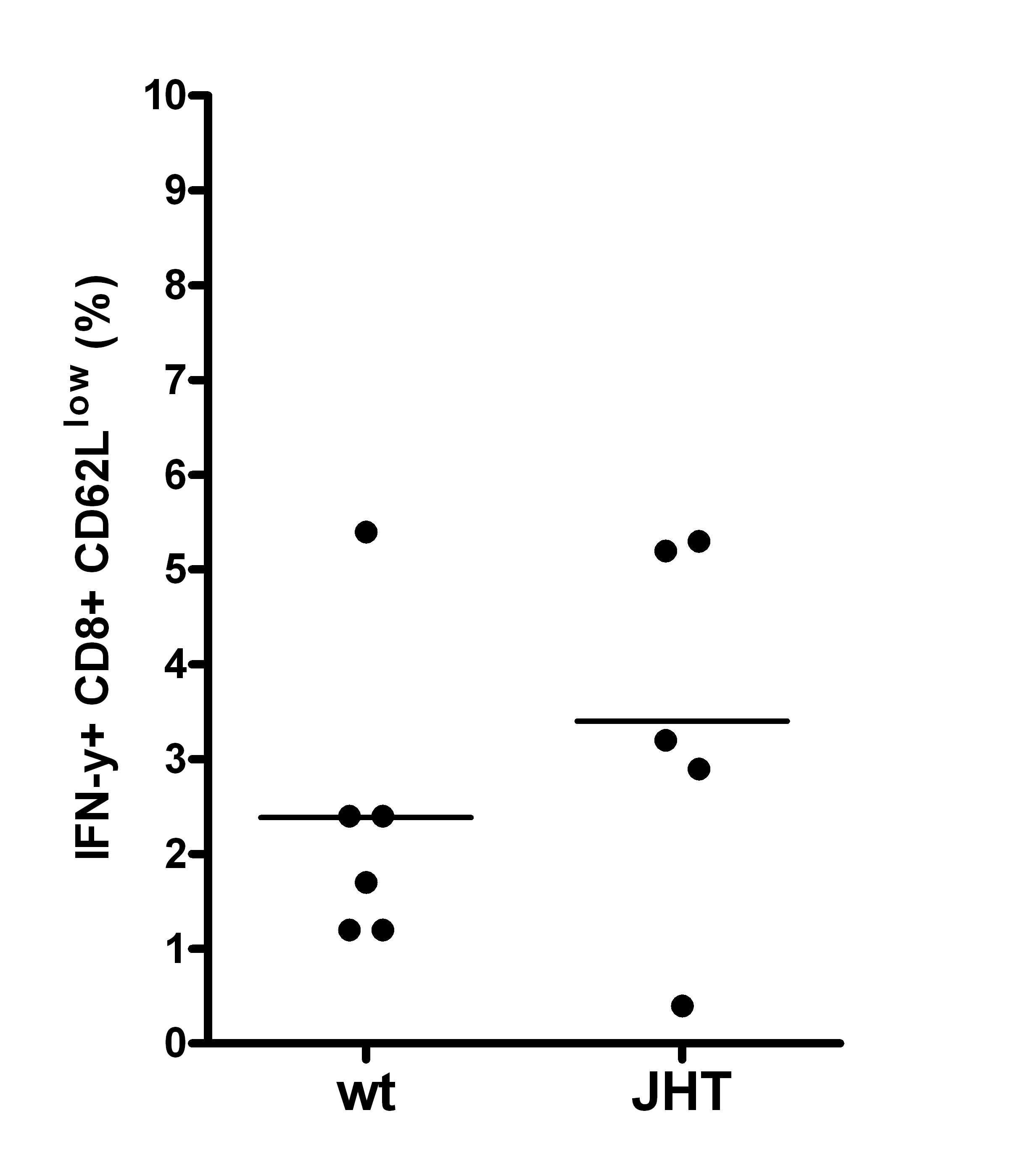

Supplement: Figure S8 — C57BL/6 mice and B cell-deficient JHT mice mount comparable VACV specific T cell responses. At 7 days after MVA immunization spleen cells from individual C57BL/6 (wt) (n = 6) mice or B-cell deficient JHT mice (n = 5) were stimulated with VACV specific peptide B8R20–27 and subsequently CD8+ CD62Llow T cells were analyzed by intracellular cytokine staining and FACS for gamma interferon expression (IFN-y+). (TIF) [file ppat.1002557.s008.tif]

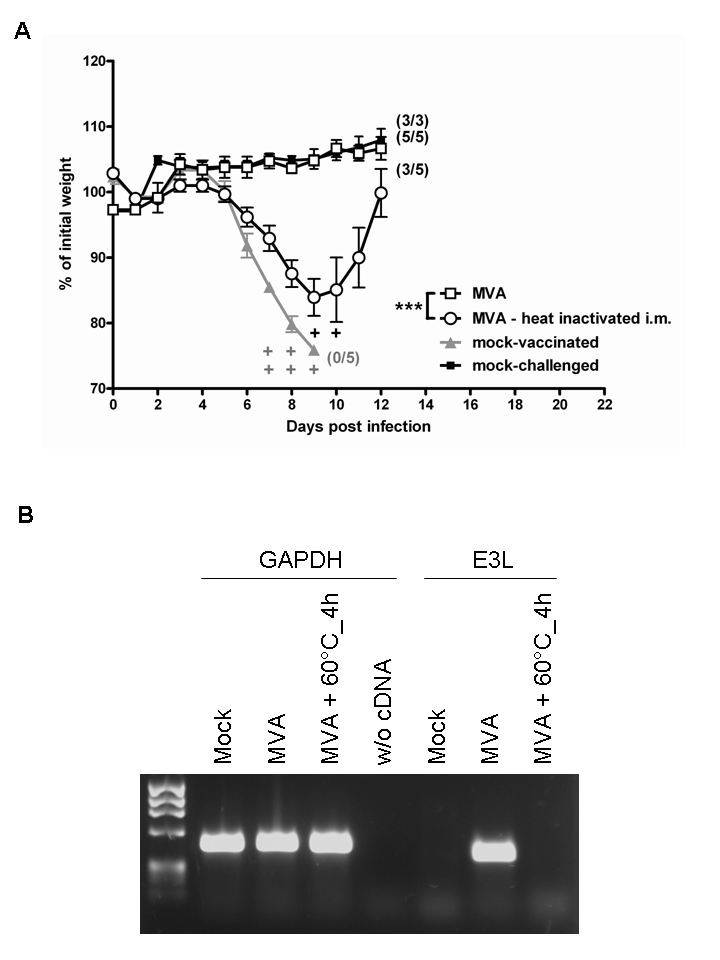

Supplement: Figure S9 — Heat-inactivated MVA vaccine does not protect from morbidity and mortality following ECTV challenge. (A) C57BL/6 mice were i.m. immunized with MVA (108 PFU) (n = 5) or heat-inactivated MVA (corresponding to 108 PFU) (n = 5) two days before 3×LD50 ECTV challenge. Mock-challenged (▪) (n = 3) and mock-vaccinated (▴) (n = 5) mice served as controls. In all experiments weight loss of individual mice was monitored daily (n = 3 to 5 per group). The data shown are representative for two similar experiments. +indicate the individual time of death. Error bars indicate SEMs, and the numbers of surviving/total animals are given in parentheses. Statistical significance of differences between groups is indicated by * for p-value<0.05, ** for p-value<0.01 and *** for p-value<0.001. (B) Confirmation of MVA inactivation. Heat-treatment (60°C for 4 hours) of MVA vaccine preparation prevents activation of viral early gene transcription. Human THP-1 cells were infected with MVA or heat-treated MVA (corresponding to an MOI of 4) and incubated for 6 h at 37°C. Total RNA was isolated from infected and mock-infected cells, and analyzed by RT-PCR using specific oligonucleotide primers for the products of VACV early gene E3L and human GAPDH. (TIF) [file ppat.1002557.s009.tif]
